# Complete Genome Sequence and Function Gene Identify of Prometryne-Degrading Strain *Pseudomonas* sp. DY-1

**DOI:** 10.3390/microorganisms9061261

**Published:** 2021-06-10

**Authors:** Dong Liang, Changyixin Xiao, Fuping Song, Haitao Li, Rongmei Liu, Jiguo Gao

**Affiliations:** 1 College of Life Science, Northeast Agricultural University, Harbin 150038, China; liangdong199082@163.com (D.L.); cecilia0920@outlook.com (C.X.); lihaitao@neau.edu.cn (H.L.); 2 State Key Laboratory for Biology of Plant Diseases and Insect Pests, Institute of Plant Protection, Chinese Academy of Agricultural Sciences, Beijing 100193, China; fpsong@ippcaas.cn

**Keywords:** *Pseudomonas*, genome, prometryne-oxidating gene, plant growth promotion

## Abstract

The genus *Pseudomonas* is widely recognized for its potential for environmental remediation and plant growth promotion. *Pseudomonas* sp. DY-1 was isolated from the agricultural soil contaminated five years by prometryne, it manifested an outstanding prometryne degradation efficiency and an untapped potential for plant resistance improvement. Thus, it is meaningful to comprehend the genetic background for strain DY-1. The whole genome sequence of this strain revealed a series of environment adaptive and plant beneficial genes which involved in environmental stress response, heavy metal or metalloid resistance, nitrate dissimilatory reduction, riboflavin synthesis, and iron acquisition. Detailed analyses presented the potential of strain DY-1 for degrading various organic compounds via a homogenized pathway or the protocatechuate and catechol branches of the β-ketoadipate pathway. In addition, heterologous expression, and high efficiency liquid chromatography (HPLC) confirmed that prometryne could be oxidized by a Baeyer-Villiger monooxygenase (BVMO) encoded by a gene in the chromosome of strain DY-1. The result of gene knock-out suggested that the sulfate starvation-induced (SSI) genes in this strain might also involve in the process of prometryne degradation. These results would provide the molecular basis for the application of strain DY-1 in various fields and would contribute to the study of prometryne biodegradation mechanism as well.

## 1. Introduction

**Figure 1:**
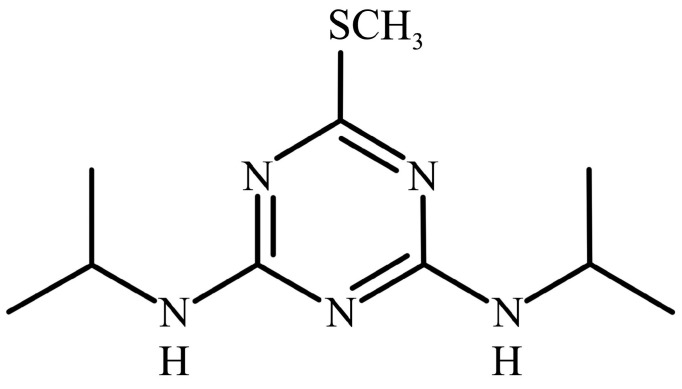
Prometryne chemical structure.

Prometryne [2,4-bis(isopropylamino)-6-(methylthio)-s-triazine] (Figure 1) is one of the most widely used herbicides of the S-triazine chemical family and be well-known use for controlling annual grasses and broadleaf weeds in modern agriculture [1]. Prometryne and its residues can be measured in significant concentrations especially in developing countries, including China, where performed a considerable quantity use of it [2]. The persistence of prometryne in soils and waters has been demonstrated by numerous studies. Its presence is detected in the environment several years after the end of use occurs [3]. Data from several studies suggest that prometryne used in soil could enter aquatic environments through adsorption, surface runoff, and rainwater erosion [4]. Correspondingly, various triazine herbicides have been detected at sampling stations in Hainan coastal waters while prometryne was detected at most stations and occurred at highest concentrations, which could indirectly affect the growth and reproduction of aquatic organisms [5]. All these lead to a series of safety problems caused by prometryne, as a total of 40% of which is applied in fields are drained into the ground polluting the aquatic environment and water supply. The others are absorbed by plants, which means they are eventually causing human health and environmental concerns by accumulating in human tissue through biomagnifications of the food chain [6].

Bioremediation is an environmentally friendly technology to efficiently degrade organic contaminants and their metabolites in water and soil [7]. However, bioremediation efficiency is limited due to the complex and toxic nature of contaminated sites and intricacies associated with the adaptation and survival of microorganisms under adverse contaminated environments [8]. Therefore, it is essential to understand the physiological, metabolic, and genetic potential of the native microorganisms in order to design a bioremediation strategy [9]. Genome sequencing combined with functional annotation is regarded as an efficient approach used in exploring functional genes which encode enzymes involved in the biodegradation of contaminants in the environment [10].

To date, only seven strains have been reported capable of degrading methylthio-s-triazine herbicides (prometryne, simetryne, ametryne, desmetryne, and metribuzin), which including *Nocardioides* sp. DN36 [11], *Arthrobacter nicotinovorans* HIM [12], *Arthrobacter* *aurescens* TC1 [13], *Nocardioides* sp. C190 [14], *Leucobacter triazinivorans* JW-1 [15], *Rhodococcus* sp. FJ1117YT [16], and *Bacillus cereus* JUN7 [17]. The first five strains are mainly using the expression products of atrazine-degrading genes such as triazine hydrolase (*trzN*), or atrazine chlorohydrolase (*atzA*), and two amido hydrolases (*atzB* and *atzC*) to transfer prometryne to the hydroxy analogues [14]. The other two strains, FJ1117YT and JUN7 perform the same prometryne degradation pathway with DY-1, which was isolated from prometryne contaminated soil in Heilongjiang Province, China. These strains degrade methylthio s-triazines via oxidation and hydrolysis, with an observation of corresponding sulfoxide, sulfone, and 2-hydroxy compounds as intermediate metabolites [16,17]. However, whole genome sequencing analysis of strain FJ1117YT and JUN7 or genes related to this degrading pathway has not been reported.

A continuous interaction is existing between plants and microorganisms in the soil ecosystem, which influences the growth, development, and functions of plants [18]. Plant growth-promoting rhizobacteria (PGPR) are the rhizosphere bacteria beneficial to plant growth by direct and/or indirect mechanisms [18]. The direct promotion includes either promoting plant nutrients uptake from environments or providing growth-promoting substances for plants. The indirect promotion occurs when PGPR prevents plants from the deleterious effects of phytopathogenic organisms [19]. The property of PGPR can be affected by the environmental conditions such as pH, soil salinity, heavy metals content, herbicides contamination, etc. Therefore, exploiting non-pathogenic rhizosphere bacteria with highly herbicide-tolerant and plant growth-promoting potential is meaningful in agriculture.

In our previous work, a novel prometryne-degrading strain of the genus *Pseudomonas* was isolated and named DY-1 [20]. Strain DY-1 has performed not only to resistant a high prometryne concentration but also to enhance the tolerance of corn against prometryne, implied its potential of promoting plant growth. The 16S rRNA gene of this strain shared 98.66% identity with *Pseudomonas resinovorans* ATCC 14235, indicating that it could be a potential novel species. In the present research, the whole genome of *Pseudomonas* sp. DY-1 was sequenced. This study intends to identify the gene involving in prometryne oxidation and to screen for the plant growth-promoting determinants in strain DY-1 through genome mining. Detailed analysis of the related metabolic pathways revealed the genetic adaptation of this strain for survival under prometryne contaminated complex conditions and the genetic basis beneficial to plant growth. Moreover, a gene encoding prometryne-degrading monooxygenase was first identified and characterized. These analyses provide the basis to further understand the mechanisms for prometryne degradation and plant growth promotion in *Pseudomonas* spp.

## 2. Materials and Methods

### 2.1. Bacterial Strains and Culture Conditions

*Pseudomonas* sp. DY-1 (hereafter, DY-1) was isolated from the soil samples collected from the top 0–20 cm of a paddy field polluted by prometryne for over five years, in Heilongjiang Province, China (46°63′75.78″ N, 126°74′87.78″ E). Prometryne removal efficiency of DY-1 was 100% after 48 h incubation, and the degrading activity of this strain can be detected even when prometryne concentration was up to 500 mg/L [20].

For prometryne-degrading-ability detected, DY-1 (or its mutant) was inoculated with 5% (*v*/*v*) into the minimal salt medium (MSM) (pH 7.0) supplemented with 50 mg/L prometryne as the sole carbon source and cultured at 30 °C in the dark on an orbital shaker at 160 rpm for 48 h. For the other analyses, DY-1 was grown in Luria Bertani medium (LB) media. *Escherichia coli* strains DH5α in the present study were used for gene cloning, while BL21 (DE3) for gene expression. *E. coli* strains were grown in LB media at 37 °C. Kanamycin (50 μg/mL) and carbenicillin (100 μg/mL) were added if necessary.

### 2.2. Genomic DNA Sequencing and Annotation

The genomic DNA was extracted by using the EasyPure^®^ Bacteria Genomic DNA Kit (TransGen Biotech, Beijing, China) according to the manufacturer’s protocol and quantified by NanoPhotometer P300 (Implen, Inc., Munich, Germany).

The purified DNA was sequenced by using the PacBio RS II sequencing platform using Single-Molecule Real-Time (SMRT) sequencing technology. After the whole genome was assembled by Hierarchical Genome-Assembly Process (HGAP, v.2.3.1), the coding sequences (CDSs) were predicted by Prodigal v.2.6 [21]. Function annotation of DY-1 was performed using the Prokaryotic Genome Annotation Pipeline (PGAP) algorithm of the National Center for Biotechnological Information (NCBI). Classification of the protein-coding genes was made using the eggNOG v.5.0 [22] database and took the best hit one as the mapping basis for Clusters of Orthologous Group (COG) annotation with an e-value threshold of 10^−5^. Annotated genes were mapped against the Kyoto Encyclopedia of Genes and Genome (KEGG) database [23] to its functional analysis. RNAmmer v.1.2 [24] and Infernal v.1.1.1 [25] were used to identify rRNA, tRNA, and ncRNA genes. Moreover, the insertion sequence (IS) elements were identified using the IS finder [26] with an e-value threshold of 10^−5^.

Transport system and function proteins analyses were performed by comparing each predicted protein against KEGG ENZYME Database [27], Pfam database [28], eggNOG database [22], transporter classification database (TCDB) [29], and the virulence factor database (VFDB) [30].

### 2.3. Phylogenetic Analysis

Based on the relationship observed from 16S rRNA alignment [20], eight type strains with different species belong to *Pseudomonas* were selected as follows: *Pseudomonas lalkuanensis* PE08 (CP043311.1), *Pseudomonas resinovorans* NBRC106553 (AP013068.1), *Pseudomonas furukawaii* KF707 (AP014862.1), *Pseudomonas aeruginosa* DSM50071 (CP012001.1), *Pseudomonas knackmussii* B13 (HG322950.1), *Pseudomonas sihuiensis* KCTC32246 (LT629797.1), *Pseudomonas oryzae* KCTC32247 (LT629751.1), and *Pseudomonas mendocina* NCTC10897 (LR134290.1).

For phylogenetic analysis of DY-1, multilocus sequence analysis (MLSA) for four housekeeping genes (16S rRNA, *gyrB*, *rpoD*, and *rpoB*) was performed [31]. The alignment for the four concatenated genes was done using ClustalX2 software [32]. Phylogenetic distances were calculated by the Jukes–Cantor algorithm, and a neighbor-joining tree was built using MEGA7 software [33] with bootstrap values were calculated in percentage from 1000 replications. Calculation of average nucleotide identity based on BLAST (ANIb) were done by JSpecies website (http://jspecies.ribohost.com/jspeciesws/, accessed 10 March 2021) [34].

The collinearity analysis was performed by BLAST+ v.2.11.0 (e-value cut-off of 10^−5^) using the genome of *Pseudomonas* sp. DY-1 genome as the query against the *P. lalkuanensis* PE08 genome. The visualization was plotted by the TBtools software [35].

### 2.4. Construction of Expression Vector

The gene encoding the monooxygenase MO5660 (WP_120651018.1) was identified from the chromosome sequence of DY-1. This gene (locus tag: D6Z43_RS05660) was amplified by polymerase chain reaction (PCR) from the DY-1 genomic DNA sample. KOD -Plus- DNA Polymerase (Toyobo Co., Ltd., Osaka, Japan) was used for all PCR reactions. The reaction system consisted of 1× -KOD- Plus buffer, 0.2 mM of dNTPs, 1 µM of MgSO_4_, 0.3 µM of primer (for each), 200 ng of genomic DNA, 1 U -KOD- Plus polymerase with a supplement of ddH_2_O up to a total volume of 50 µL. Amplification was performed for 35 cycles after pre-denaturation at 94 °C for 2 min. Cycling conditions were set to denaturation at 94 °C for 15 sec, annealing at 59 °C for 30 sec, and extension at 68 °C for 90 s. The amplification product was analyzed by electrophoresis on 1% (*w*/*v*) agarose gel and purified using Axygen^®^ DNA Gel Extraction Kit (Axygen Biosciences, Union City, CA, USA) following the manufacturer’s instruction. DNA sequences of the oligonucleotides used in this study are listed in Table S1.

Restriction digestion was performed on pET28a at *Xho* I and *Sal* I sites to obtain linearized plasmid. The insert fragment and the linearized pET28a were blunted by using ClonExpress^TM^ II One Step Cloning Kit (Vazyme, Nanjing, China). The recombinant plasmid was then transformed into *E. coli* DH5α through the heat-shock method, and the recombinant strain was incubated on solid LB medium (contained kanamycin) at 37 °C for 12–16 h. The verification of successfully constructed plasmid was performed via Colony PCR and sequencing.

### 2.5. Gene Expression

The recombinant plasmid was extracted using Axygen^®^ Plasmid Miniprep Kit. After then, a second transformation was carried out in *E. coli* BL21 (DE3) using the same method as above. Plasmid pET28a without insert fragment was used as a control in downstream experiments. The obtained transformant was inoculated into 5 mL of LB medium supplemented with kanamycin and pre-cultured overnight at 37 °C, 220 rpm. These liquid cultures were then inoculated with 1% (*v*/*v*) into a 500 mL flask containing 100 mL of LB medium supplemented with kanamycin and incubated at 37 °C, 220 rpm to an optical density at 600 nm (OD_600_) of 0.6. For inducing expression, isopropyl-β-D-thiogalactopyranoside (IPTG) was added into the cultures with a final concentration of 0.5 mM. After induction for 16 h at 16 °C, 160 rpm, cells were harvested by centrifuging at 4000× *g* for 10 min at 4 °C and washed at least twice by 10 mM phosphate buffer saline (PBS, pH 7.4). The cells were disrupted by ultrasonic treatment in an ice-water mixture for 10 min of 3 s, with 5 s intervals. Debris and unbroken cells were removed by centrifuging twice at 12,000× *g* for 15 min at 4 °C [36]. The supernatant was filtered through a 0.22 µm filter and purified using Ni-NTA Fast Start Kit (Qiagen, Hilden, Germany) according to the instructions in the manual. After purification, FAD was added in the purified protein with a final concentration of 10 µM.

The samples of purified products and control were mixed with 2 × SDS loading buffer [100 mM Tris-HCl pH 6.8, 200 mM β-mercaptoethanol, 4% (*w*/*v*) SDS, 2% (*w*/*v*) bromophenol blue, and 20% (*v*/*v*) Glycerol], treated with boiling water bath for 5 min and centrifuged at 10,000× *g* for 10 min. The supernatant was collected for protein separation using 10% SDS-PAGE and visualized by Coomassie stain. Protein concentration was determined using bovine serum albumin (BSA) as a standard following the user manual of the BSA Protein Assay Kit (TransGen Biotech, Beijing, China).

### 2.6. Analysis of Prometryne-Degrading Capacity

To test the prometryne-degrading activity of purified MO5660, closed Eppendorf tubes containing 300 μM of prometryne, 60 μM of NADPH, and 0.032 mg/mL of enzyme solution in PBS, were incubated at 30 °C for 1 h. Eppendorf tube with the enzyme solution was replaced by induced pET28a-BL21 (DE3) suspension was set as a control. Prometryne extraction was in accordance with those described in Liu et al. [15] with minor modification. Briefly, the culture mixtures were shaken with an equal amount of dichloromethane as the extractant and let stand at room temperature. The organic phase was evaporated in a rotary evaporator and dissolved in 1 mL of methanol. The extracts were then filtered by 0.22 μm organic membrane and analyzed by high efficiency liquid chromatography (HPLC ) using an Agilent TC-C_18_ column (Agilent Technologies, Santa Clara, CA, USA). The mobile phase was methanol/water (80:20, *v*/*v*) with a flow rate of 1 mL/min at 25 °C [37]. Prometryne was monitored at 216 nm. The amount of prometryne in the samples (peak area) was calculated by comparing it with that of a known amount of reference standard. The degradation rate of prometryne was calculated using Equation (1):

((C_0_ − C_t_)/C_0_) × 100% (1)

where C_0_ and C_t_ are the concentrations of prometryne at time 0 and time t, respectively. Each experiment was repeated three times. The average of three measurements with a standard deviation was calculated to plot the graphs.

Effects of important parameters, i.e., culture temperature and pH value, on the prometryne-degrading ability of purified MO5660 were investigated. The optimal temperature was determined at pH 7.0 in the range 20–50 °C in intervals of 5 °C. The optimal pH was determined at 30 °C using Britton-Robinson buffer [38] over a pH range of pH 4.0–10.0 in intervals of 1.0. All assays were performed using the method described above. Each treatment was performed in three replicates.

### 2.7. Gene Knock-Out

To further verify whether the *mo5660* gene is necessary for DY-1 to degrade prometryne, this gene was replaced with kanamycin resistance cassette (*kanR*) using the λ-Red recombinase system. The plasmid containing λ-Red operon was constructed as described by Lesic et al. [39], with minor modifications. The λ-Red operon fragment was amplified from the pKD46 plasmid by PCR with primers RedF/RedR, which contained *Kpn* I and *Hind* III restriction sites. PCR conditions were 2 min at 94 °C followed by 30 cycles of 15 sec at 94 °C, 30 sec at 60 °C, and 3.5 min at 68 °C. After purification, this fragment was digested with *Kpn* I and *Hind* III. The pUCP18 plasmid [40] was linearized by the same enzymes. Both the fragment and plasmid were purified from agarose gel then the two products were ligated using T4 DNA ligase to construct the vector pUCP18-Red.

To generate the electro-competent cells of DY-1, the single colony was inoculated in 5 mL of LB medium and incubate overnight. This culture was inoculated with 5% (*v*/*v*) in 100 mL of LB medium and incubate to an OD_600_ of 0.5–0.6. The cells were harvested after centrifuging at 6000× *g* for 5 min at 4 °C, followed by three times washing using double volume ice-cold 10% (*w*/*v*) glycerol. The cell pellet was subsequently resuspended in 1 mL of ice-cold 10% (*w*/*v*) glycerol, transferred to a 1.5 mL centrifuge tube, and centrifuged at 6000× *g* for 5 min at 4 °C. One hundred micro-liters of ice-cold 10% (*w*/*v*) glycerol was then added to resuspend the pellet. The mixture of 100 μL of bacterial suspension and no more than 10 μL of DNA in a 0.2 cm ice-cold electroporation cuvette was electroporated by GenePulser Xcell (Bio-Rad, Hercules, CA, USA) with the parameters set as 2.5 kV (12.5 kV/cm), 25 μF, and 200 Ω. Nine hundred micro-liters of SOC medium were immediately added into the cuvette and gently mix with the cells after electroporation. The cells were transferred to a 2.0 mL centrifuge tube and incubate at 30 °C for 2 h and centrifuge at 12,000× *g* for 1 min at room temperature. After then, a total of 900 μL of the supernatant was discarded, cells were resuspended in the residual medium and spread on an LB plate containing carbenicillin. The plate was incubated at 30 °C until colonies appeared [41].

After overnight incubation, transformants were randomly picked and verified by digestion of enzyme *Hind* III and sequencing.

**Figure 2:**
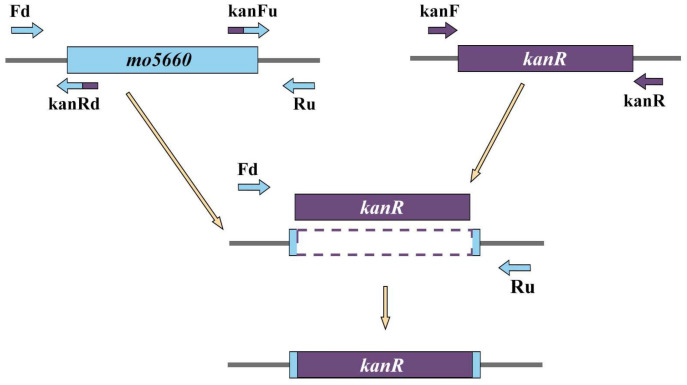
Construction of *mo5660* knock-out mutant by gene replacement. First step: Upstream and downstream regions of *mo5660* were amplified independently along with the kanamycin resistance cassette (*kanR*) which was amplified from pKD4 plasmid with primers KanF and KanR. The 3′ end of kanRd and kanFu primers had forty nucleotides homologous to *mo5660* while the 5′ end had twenty nucleotides homologous to *kanR*. Second step: PCR products obtained from the first step were mixed together in equal concentration, amplified with primers Fd and Ru to generate the Δ*mo5660*.

Using the vector pKD4 as the template, a cassette encoding kanamycin resistance was amplified with the primers kanF/kanR. Primers Fu/kanRu and kanFd/Rd were designed to amplify upstream and downstream regions of the target gene, and to allow the 5′ and 3′ ends of the gene unmodified in the recombinant mutant (Figure 2).

The two approximately 60 bp primers among these four, kanRu and kanFd, contained a 20 bp-region homologous to 5′ end of the kanamycin-resistant cassette (kanR). The regions flanking of the target gene were amplified from DY-1 genomic DNA with the primers Fu/kanRu and kanFd/Rd. After the three fragments (*kanR* and the regions flanking the target gene) were obtained, 25 ng of each were mixed to amplify the recombinant fragment with the primers Fu/Rd. Thermocycling conditions are as described in Section 2.5 except those annealing temperatures were 55 and 60 °C for the gene cassette and the flanking regions respectively.

Recombinant strain DY-1/pUCP18-Red was cultured in LB medium containing carbenicillin to OD_600_ of 0.4 and induced for 2 h by 0.2% (*v*/*v*) L-arabinose. Afterwards the bacteria were treated as described above, and the purified recombinant fragment was transferred into DY-1/pUCP18-Red by electroporation. The final culture was spread on an LB plate containing carbenicillin and kanamycin. Colonies were verified via PCR with the two sets of primers outF/kanRin and outF/outR followed by sequencing.

To assay the prometryne-degrading capacity, the overnight cultures of both DY-1 and Δ*mo5660* were inoculated into MSM medium containing prometryne and cultured for 48 h. Samples were collected every 2 h. HPLC was used to detect the residual concentration of prometryne.

### 2.8. Bioinformatic Analysis of MO5660

Proteins homologous to ethionamide monooxygenase (EthA), cyclohexanone monooxygenase (CHMO), cyclopentanone monooxygenase (CPMO), cyclopentadecanone monooxygenase (CPDMO), 4-hydroxyacetophenone monooxygenase (HAPMO), phenylacetone monooxygenase (PAMO), and MO5660 were identified in both the NCBI NR and SwissProt databases [42], the accession number of each sequence considered in the alignment is given in Table 1. Subsequently, a multiple sequence alignment of the resulting 63 Baeyer-Villiger monooxygenase (BVMO) sequences was created with MEGA7 software using the Muscle algorithm [33]. Following that, the phylogenetic tree was created using the neighbor-joining method with 1000 bootstrap replicates and visualized using the Tree of Life (iTOL) (https://itol.embl.de/, accessed 15 March 2021) online tool [43].

The physico-chemical properties analysis and secondary structure prediction of MO5660 were done by the ProtParam online server (https://web.expasy.org/protparam/, accessed 27 February 2021) [44]. The online servers TMHMM v.2.0 (http://www.cbs.dtu.dk/services/TMHMM/, accessed 15 March 2021), SOSUI (https://harrier.nagahama-i-bio.ac.jp/sosui/sosui_submit.html, accessed 15 March 2021) [45], and SignalP v.5.0 (http://www.cbs.dtu.dk/services/SignalP/, accessed 15 March 2021) [46] were used to predict transmembrane regions and signal peptides.

## 3. Results

### 3.1. Genomic Features

**Figure 3:**
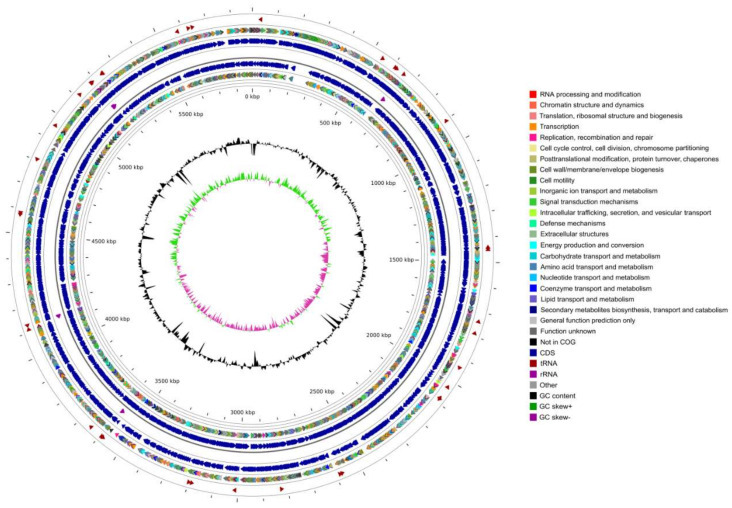
Circular genome map and features of *Pseudomonas* sp. DY-1. The contents of the featured rings (starting with the outermost ring to the center) are as follows. Ring1: tRNA-related genes; Ring2: the COG annotation gene distribution of the positive strand, distinguished by different colors; Ring3: genes position of the positive strand; Ring4: rRNA genes; Ring5: genes position of the negative strand; Ring6: COG annotation of the negative strand; Ring7: GC content, taking the average GC as the baseline, the outward protruding means higher than the average, and the inward protruding means lower than the value; Ring8: the GC skew (G − C/G + C) values, values < 0 in purple and values > 0 in green.

The genome of *Pseudomonas* sp. DY-1 was sequenced and generated 122,499 filtered sub-reads with approximately 250-fold coverage and a mean read length of 5809 bp from one SMRT cell. The assembled genome consists of a single circular 5.89 Mb chromosome (NZ_CP032616.1) and a circular plasmid named DY-1p1 (NZ_CP032615.1, 26,350 bp). The complete genome contains 5543genes corresponding to 5452CDSs, 91 RNA genes, and 75pseudogenes. GC content of the whole genome was 62.94%, the main genomic features are summarized in Table 1. The circular genome map of DY-1 shows the genome distribution (Figure 3).

### 3.2. Phylogenetic and Collinearity Analysis

**Figure 4:**
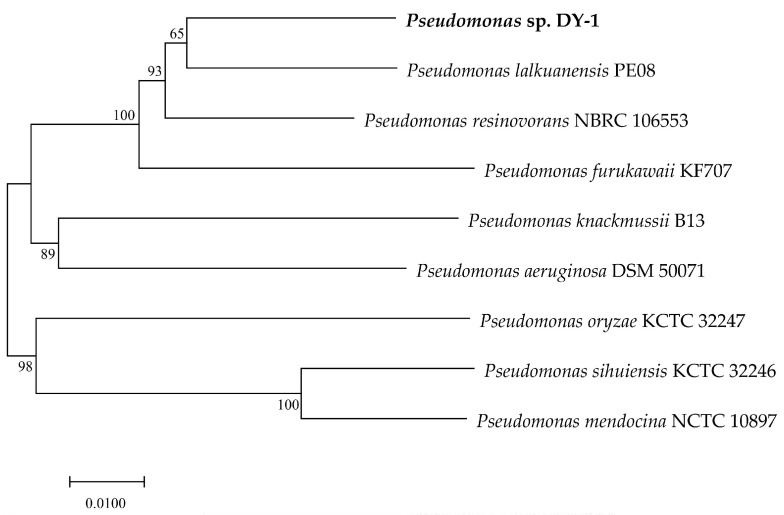
Phylogenetic tree based on the 4-genes MLSA for *Pseudomonas* sp. DY-1 and other eight type strains. The distance matrix was calculated by the Jukes-Cantor method. The dendrogram was generated by neighbor-joining. Branches corresponding to partitions reproduced in more than 50% bootstrap replicates (from 1000 replicates) are shown with the bootstrap value at the nods.

Figure 4 displays the phylogenetic tree constructed based on MLSA, which shows *Pseudomonas* sp. DY-1 is most closely related to *P. lalkuanensis* PE08 (CP043311.1) with a similarity value of 94.78%. The ANIb between DY-1 and PE08 is 87.12% (Table S1). Both two values are below the cut-off (96% for MLSA and 95% for ANIb) for shared species identity. Thus, DY-1 might not be assigned to any currently known species of the genus *Pseudomonas* available in the NCBI database.

The collinearity analysis of the DY-1 genome against the two closest PE08 and NBRC 106553 genomes was performed (Figure S1). It can be seen that DY-1 is closer to PE08 than NBRC 106553, which is the same as Figure 4 shows. However, the range of gene rearrangement was relatively wide and there was a large area of gene location transfer. This result implies that the core genome is conserved while the accessory is divergent, which may lead to the evolution of the strain and variation in genotypes.

### 3.3. Database Annotation

#### 3.3.1. VFDB Analysis

Virulence and toxin encoding genes associated with safety were evaluated in the genome of strain DY-1 using VFDB [30] with threshold coverage of >70%, percent similarity > 70%, and E-value < 0.0001. The results suggest that virulence factors related to genus *Pseudomonas*, including factors involved in regulating, secreting, pigment and biosurfactant synthesizing, protease, and toxin encoding, are not detected in strain DY-1.

#### 3.3.2. COG Analysis

The COG codes were classified based on the annotated function of protein-coding genes of *Pseudomonas* sp. DY-1 (Figure 2 and Figure S2). Of the 5452 protein-coding genes, a total of 4497proteins were assigned. Among the metabolism of COG categories, the four most abundant COG types were amino acid transport and metabolism (553, 10.27%), energy production and conversion (356, 6.61%), lipid transport and metabolism (286, 5.31%), inorganic ion transport and metabolism (276, 5.13%), and carbohydrate transport and metabolism (239, 4.44%). Proteins for nuclear structure (Y) and cytoskeleton (Z) were not detected. The least number of genes were annotated in the categories of RNA processing and modification (2, 0.04%) and chromatin structure and dynamics (3, 0.06%). The function of more than 37% of the total protein remained unknown, including 574 proteins of “general function prediction only” and 352 of “function unknown”, which might imply many proteins with degrading function has not yet to be identified.

#### 3.3.3. KEGG Analysis

A total of 1608 out of the 5452 protein-coding genes were annotated into 40 biological pathways in the KEGG database. As shown in Figure S3, most of the annotated genes participate in the category of “metabolism”, which was more than any other category. Among “metabolism”, “amino acid metabolism” (348, 10.41%), “carbohydrate metabolism” (245, 7.33%) and “energy metabolism” (198, 5.92%), and “metabolism of cofactors and vitamins” (184, 5.5%) were the four types with the largest number of annotated genes.

### 3.4. Molecular Characteristics of Pseudomonas sp. DY-1 Genome

#### 3.4.1. Carbohydrate Metabolism

Inspection of the genome of DY-1 reveals that it encodes all the enzymes of not only the canonical Entner-Doudoroff (ED), pentose phosphate (PP), and Shikimate pathways, but gluconeogenesis, as well tricarboxylic acid (TCA) and glyoxylate cycles (Table S4). The carbohydrate metabolism system of DY-1 consists of these pathways. It can be observed that the enzyme system involved in the glycolytic pathway (EMP) is incomplete because of a missing 6-phosphofructokinase (PFK). It is thus likely that similar to most *Pseudomonads* strains [47,48], DY-1 utilized glucose exclusively via the ED pathway enzymes. Although the EMP used by many organisms is considered to be the predominant textbook route for the metabolism of glucose, in reality, the ED counterpart is the most frequent biochemical device found in environmental bacteria and archaea [49]. The ED route is more proficient in the generation of reducing power than EMP, in particular NADPH [50], which is required not only for anabolic functions but also for counteracting different types of environmental stress [51]. Therefore, this pathway contributes to the physiological heftiness of environmental bacteria in their natural habitats via gearing their aerobic metabolism tolerance to oxidative stress from both in- and outside [49].

#### 3.4.2. Nitrogen Metabolism

Nitrogen plays a major role in the metabolism of rhizosphere-dwelling *Pseudomonas* spp., especially through nitrate or nitrite reduction [52]. The genes encoding nitrite reductases (NirB and NirD) were identified in the DY-1 genome (Table S5). These two genes have also been found in many rhizosphere-associated *Pseudomonas* spp. [53,54] and their roles in rhizocompetence have often been confirmed [55].

#### 3.4.3. Sulfur Metabolism

Sulfur is another essential element for microbes and plants [56]. The preferred source of sulfur for bacteria is that for cysteine biosynthesis present in the form of inorganic sulfate [57] putative genes related to this pathway in DY-1 are listed in Table S6. The sulfate molecule is actively transported into the cell by an ABC-type transporter, activated to phosphoadenosine-phosphosulfate, and reduced via sulfite to sulfide. Sulfide is then added to a carbon chain by trans-sulfurization, by other pathways to give cysteine [57]. However, sulfur present in both agricultural and uncultivated soils is largely in the form of sulfate esters and carbon-bonded sulfur (sulfonates or peptide residues) [58]. Thus, the ability to utilize organosulfur compounds is critical for bacteria survival in the soils [59]. In response to the limited availability of inorganic sulfur sources, many soil bacteria perform to assimilate organic sulfur sources mediated by the sulfate starvation-induced (SSI) stimulation [60]. Similar to that in *P. putida* S313 [59], DY-1 contains the *ast-sft* gene cluster encoding enzymes involved in the utilization of sulfate (Table S6) [61]. In addition, two systems for sulfonate utilization are identified (Table S6). The *cysP* gene encodes the periplasmic sulfate-binding protein and is encoded together with *cysTWA*, the remaining components of the sulfate-transport system. This genetic organization is consistent with that observed in *E. coli* [62]. The *tauABCD* operon, which is required for the utilization of taurine as a sulfur source [63], was also observed in DY-1. Methanesulfonate is a natural oxidation product of dimethyl sulfide; it is the main biogenic organic sulfur compound in the atmosphere and is present in significant quantities in rainwater [64]. The *msuD* and *msuE* genes were found in the DY-1 genome. In *P. aeruginosa* PAO1, these two genes encode a methanesulfonate-specific FMNH_2_-dependent sulfonatase and an FMN reductase, respectively [64].

Genome mining also revealed the potential of DY-1 for detergent biodegradation. Two genes involved in sodium dodecyl sulfate (SDS) biodegradation were observed. One of these, *sdsA*, codes for an alkyl sulfatase, while the another, *sdsB*, codes for a positive activator protein [65].

#### 3.4.4. Potential of Plant Growth Promoting

As it shows in Table S7, genetic determinants involved in plant growth promotion in DY-1 include the 1-aminocyclopropane-1-carboxylate (ACC) deaminase coding gene, the product of which contributes to reduce ethylene production and enhance plant growth by cleaving the precursor of plant ethylene precursor (ACC). The mechanism mediated by this gene has been observed in PGPR [66]. Riboflavin stimulates plant growth and is known to function as a protectant/elicitor of plant defense [67]. DY-1 may be capable of helping plants against the pathogenic fungi and bacteria since its genome encodes complete biosynthesis operons for riboflavin (Table S7).

Alginate, one of the extracellular polysaccharides mainly produced by *Pseudomonas* strains, can not only supply the additional survival advantages for the organism but contribute to colonization and biofilm persistence [68]. In addition, alginate production contributes to the surface colonizing of plants [69]. The previous study has portrayed that the structure and adherent nature of cells in biofilms modulate biocontrol activities and antimicrobial tolerance [70]. In addition, biofilmed *Pseudomonas* spp. inoculate possess N_2_-fixing properties and nutrient uptake [71]. Gene cluster comprising twelve genes which responsible for alginate biosynthesis are present in two copies each in chromosome genome of DY-1, which might help in survival in the harsh soil environment and enhance the rhizosphere colonization ability.

To adapt, survive and reproduce successfully in nature, all organisms, including host plants and microbes, release chemicals that serve as hostile or friendly signals [72,73]. These signals can be delivered as soluble compounds or volatiles [74]. It has been demonstrated that bacterial volatiles played a role as a bacterial protectant. A gene encoding 2,3-butanediol dehydrogenase was detected in the DY-1 chromosome, suggested that this gene might involve in the synthesis of 2,3-butanediol, one of the well-characterized bacterial volatiles with the antimicrobial activity. It showed that DY-1 had the potential to resist plant pathogens such as *Erwinia carotovora pv. carotovora*, *Colletotrichum orbiculare*, *Rhizoctonia solani*, and *Sclerotinia homoeocarpa* by synthesizing volatiles [74].

Competition for iron is exacerbated in the rhizosphere because it is an essential element for the primary metabolism of most organisms [75]. Plant beneficial bacteria are able to efficiently compete for iron in the rhizosphere to inhibit microbial rivals. This competition can lead to high inhibition levels against plant pathogens, making iron starvation to become a biocontrol mechanism against fungi [76]. Similar to many microbes [77], besides the ABC-type transport systems, DY-1 is likely able to secrete low-molecular iron-chelating compounds (siderophores) to gain access to iron (Table S7).

#### 3.4.5. Environmental Stress Resistance

Adaptation to environmental stresses, such as temperature fluctuation, is essential for the survival of all living organisms [78]. Cold shock protein (Csp) can help protein folding at low temperatures [79]. Besides, the bacterial *cspA* genes can enhance maize and wheat resistance to drought stress and improve the grain yield [80]. Genomic insight into temperature adaptation illustrated the presence of several copies of cold shock proteins coding genes (Table S8). These characteristics of DY-1 may contribute to the drought tolerance of plants.

Reactive oxygen species (ROS), such as superoxide and H_2_O_2_, are produced and accumulated in organisms along with aerobic metabolism [81]. Bacteria produce many antioxidant enzymes to enhance their tolerance to ROS [82]. The genome information of DY-1 shows that this strain may respond to these adverse effects by synthesizing antioxidant enzymes such as thioredoxin, peroxidase, and superoxide dismutase (Table S8). In addition, alkyl hydroperoxide reductase (Ahp) encoded by the DY-1 chromosome is likely to be the primary scavenger of endogenous H_2_O_2_. It has been reported that Ahp in *E. coli* was a more efficient scavenger of trace H_2_O_2_ than catalase [83].

As shown in Table S8, DY-1 possesses the genes which encoded proteins associate with osmoregulation and pH homeostasis. Among these products, NhaA, NhaB, NhaP, and NhaP2 proteins are sodium ion/proton antiporters that use the proton electrochemical gradient to expel sodium ions from the cytoplasm and functions primarily in the adaptation to high salinity at alkaline pH [84]. C4-dicarboxylate carriers catalyze the uptake of C4-dicarboxylates by H^+^/Na^+^ symport, which plays a role in the regulation of pH homeostasis and the sodium cycle of bacteria [85]. Besides, the Na^+^ cycle plays an important role in pH homeostasis, the capacity for the latter directly determines the upper pH limit of bacteria growth [86]. Another system encoded by *mrp* and *pha* genes, which was first identified in *Bacillus halodurans* for pH homeostasis [87], was also observed from the chromosome of DY-1. Moreover, the genome of DY-1 also contains genes encoding potassium uptake proteins (Trk) and mechanosensitive channels (MscL and MscS) (Table S8), which make the strain able to grow at low osmotic pressure [88]. These results suggest that DY-1 possesses an effective osmoregulation system to adapt to harsh environments.

Cyanide is highly toxic for most living organisms because it can form stable complexes with transition metals which are essential for protein function [89]. The presence of cyanide in the environment can lead to the unavailability of the metals essential for the organisms. It has been reported that iron acquisition and oxidative stress are related processes that can be grouped as resistance mechanisms [90]. The *cio* cluster and *mqo* gene have been demonstrated to be correlated with cyanide metabolism [90]. The cyanide degradation pathway in some bacterium requires malate: quinone oxidoreductase that converts malate into oxaloacetate, which reacts chemically with cyanide, forming a cyanohydrin which is further hydrolyzed by nitrile hydratase, generating ammonium [91,92]. Since the corresponding genes were all identified in the DY-1 genome, this strain likely had a certain resistance to cyanide and might be capable of assimilating cyanide as well.

#### 3.4.6. Metal/Metalloid Transport and Resistance

It has been revealed that the interactions between plants and microbe play a notable role in enhancing metal phytoextraction. Previous studies have reported that plant-associated microorganisms contribute to the mobilization and uptake of heavy metals by plants [93] as well as the improvement of plant growth under metal exposure [93].

As shown in Table S9, the genome of DY-1 carries many typical copper resistance genes, such as *czcC*, *cotA*, *copASRBZ*, etc. Besides, it also carries genes involved in the transport and resistance of chromate, tellurite, arsenate, such as *chrAB*, *tehAC*, *arsCBH*, etc.

Copper and zinc are essential trace elements for many organisms, while also can be toxic when they are in excess [94]. Through the information of the DY-1 genome, the copper resistance and detoxification in DY-1 could be achieved via oxidation and efflux mechanism, while that for zinc mainly based on efflux mechanism [95].

As one of the broadest ranges used of elements, the beneficial influence of chromate in the biosphere is unknown [96]. Strategies for chromate stress against bacteria are mostly based on chromate efflux mechanism and reduction reaction of Cr (VI) to Cr (III) [97]. Referring to the genome annotation analysis DY-1 possesses a putative *chrA* gene, which is regulated by the product of *chrB*, may be responsible for chromium tolerance [97]. Therefore, DY-1 may detoxify chromate mainly via the ChrA-mediated efflux process.

#### 3.4.7. Polymer Biosynthesis

Polyhydroxyalkanoate (PHA) is a kind of biodegradable polymers and a great substitute for conventional petrochemical-based plastics [98]. Under the condition of limited nutrition, some bacteria can produce 3-hydroxyacylcoenzyme, the substrate for PHA production, through hydrolysis of the excessive carbon source and β-oxidation of free fatty acids. Following that, PHAs act as carbon and energy reserve materials accumulating as discrete, water-insoluble inclusions in bacterial cells [99]. A gene cluster encoding enzymes for poly(3-hydroxyalkanoate) biosynthesis was noted in the DY-1 genome (Table S10). *P. oleovorans* GPo1 has been demonstrated to accumulate PHA when alkanes or alkanoic acids were provided as carbon sources [100]. The *pha* gene cluster, which has been identified contributes to PHA biosynthesis in *P. oleovorans* GPo1 [100], was also found in the DY-1 genome (Table S10). This gene cluster encodes the proteins consists of four open reading frames (ORFs) which are transcribed in the same direction (*phaC2*, *phaZ*, *phaC1*, and *phaD*). The *phaF* and *phaI* genes located downstream of the *phaC1ZC2D* gene cluster are granule-associated proteins (GAPs), mainly responsible for preventing excessive aggregation of PHA granules and non-specific attachment of other proteins or hydrophobic molecules during metabolism [101]. These results suggest that DY-1 has the potential to be the source of PHAs.

#### 3.4.8. Xenobiotics Biodegradation and Metabolism

The homogentisate pathway, the protocatechuate and catechol branches of the β-ketoadipate pathway, and the phenylacetate pathway are the four common pathways for the degradation of aromatic compounds [102]. Genome analysis showed that expectations of the last, these pathways are existing in DY-1 (Table S11). A variety of aromatic compounds can be metabolized under aerobic conditions into protocatechuate or catechol. These central intermediates can be degraded further in several routes, which depend on the enzymes that open the ring structure. In DY-1, enzymes catalyzing ring cleavage could be protocatechuate 3,4-dioxygenase (encoded by *pcaH* and *pcaG*) and catechol 1,2-dioxygenase (encoded by *catA*), which proceed via 3-oxoadipate (β-ketoadipate) to acetyl-CoA and succinyl-CoA respectively. Moreover, gene clusters in DY-1 encoding the enzymes involved in the degradation of 4-hydroxyphenylacetate (4-HPA) are listed in Table S11. These genes here are located in two operons. The first operon consists of *hpaRBCA*, products of *hpaB* and *hpaC* gene belong to the two-component nonheme flavin-diffusible monooxygenase (TC-FDM) family. The reductase component encoded by *hpaC* uses NAD(P)H to catalyze the reduction of a flavin that diffuses to the oxygenase component encoded by *hpaB* for oxidation of the substrate by molecular oxygen [103]. Phenylacetate is a major intermediate in the bacterial degradation of many aromatic compounds [104]. The first step of phenylacetate oxidization in strain DY-1 is the activation of phenylacetate to phenylacetyl-CoA by PaaK. Then oxygen is introduced into the aromatic ring of phenylacetyl-CoA by a multicomponent oxygenase PaaABCDE [105]. Phenylacetyl-CoA is isomerized, and the ring is cleaved by PaaG and PaaZ respectively. Following that are isomerization and cleavage reactions catalyzed by PaaG and PaaZ. Finally, PaaJ, PaaF, and PaaH catalyze a complete conversion of the ring-opened intermediate successively, leading to the formation of acetyl-CoA and succinyl-CoA, the intermediates of the TCA cycle [105].

In addition to the above, 55 genes involved in the pollutant degradation were also identified in the DY-1 genome (Figure S12).

Aldoximes are intermediates in the biosynthesis of biologically active compounds such as indoleacetic acid, and cyanogenic glucosides in plants [106]. Nitrile compounds, which are toxic and hazardous compounds for all organisms, are often discharged into the environment in industrial wastewater and agricultural chemicals [107]. Genes whose products are aldoxime dehydratase, nitrile hydratase, and amidase are identified in the DY-1 genome. Enzymes encoded by these genes have been reported capable of degrading nitrile [108]. Therefore, DY-1 may help in the treatment of nitrile pollution.

Azo dyes, the most important group of synthetic colorants, are generally considered as xenobiotic compounds that are very recalcitrant against biodegradative processes [109]. Four copies of genes encoding azoreductase (AzoR) are present in DY-1. This enzyme catalyzes reductive cleavage of azo groups under mild conditions; thus, this enzyme is suggested to contribute to the development of azo dyes biodegradation [110].

Two copies of dienelactone hydrolase (DLH) coding genes in the DY-1 chromosome were observed, and they might be induced in response to the uptake of otherwise toxic halogenated aromatic compounds [111]. This enzyme is related to the transformation of toxic aromatics into innocuous citric acid cycle catabolites. Therefore, microorganisms with genes encoding DLH would serve a vital ecological role in the biosphere [112].

As it shows in Table S12, genes encoding alpha-ketoglutarate-dependent dioxygenase (AlkB), alcohol dehydrogenase (AdhC), and aldehyde dehydrogenase (ALDH) were identified in the DY-1 genome. As a key enzyme of alkane degradation, AlkB plays an important role in detoxifying alkane contaminants [113], while the products of genes *adhC* and *ALDH* were reported responsible for the degradation of various organic compounds, including alkane, chloroalkane, chloroalkene, naphthalene, and methane [114]. Thus, the existence of these genes revealed the potential of DY-1 for application in bioremediation of hydrocarbon contaminated sites.

### 3.5. Sequence Analysis of MO5660 Monooxygenase

A putative monooxygenase coding gene (named *mo5660*) was noted since the result of BLASTp against UniProtKB/Swiss-Prot database [42] showed its product, MO5660, shared a 53% sequence identity and 69% sequence similarity with EthA of *Mycobacterium tuberculosis* H37Rv, which was responsible for the oxidation of the pro-drug ethionamide into its bioactive form [115]. The cloned *mo5660* gene was 1530 bp in length with a GC content of 64%, encoded a protein of 509 amino acids with a calculated molecular mass of 56444.62 Da. Signal peptide and transmembrane regions were not found in protein sequence.

Upon sequence alignment (Figure S4), the typical consensus sequences for type I BVMOs, two BVMO fingerprint motifs flanked by two Rossmann fold motifs [116], were identified in MO5660.

**Figure 5:**
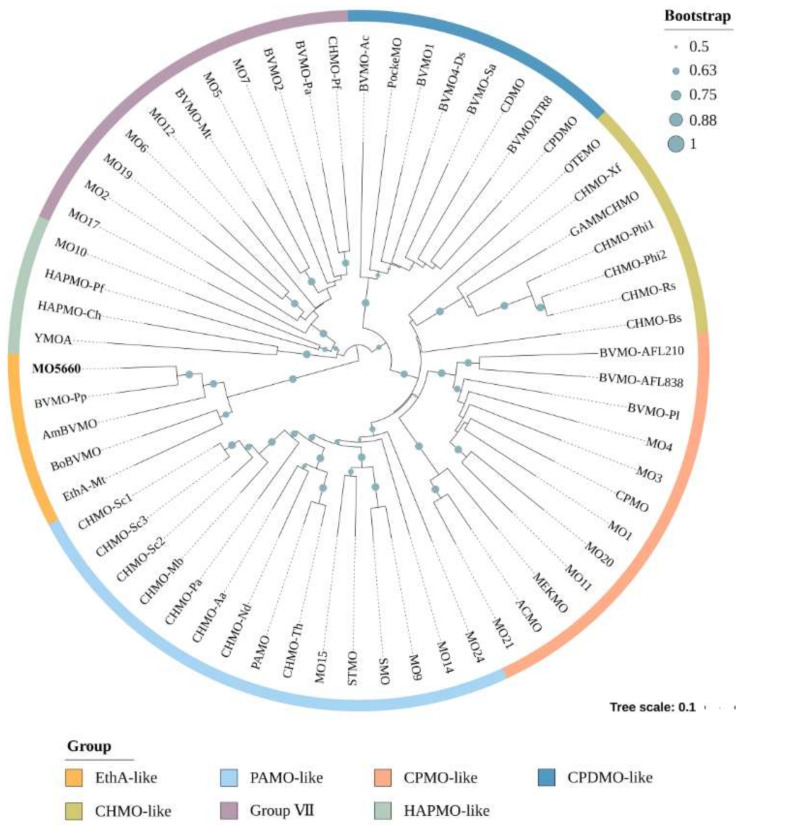
Phylogenetic tree including MO5660. Bootstrap values of more than 500 (from 1000 replicates) are marked with circles of different sizes.

To establish the evolutionary relationship of MO5660 with other reported BVMOs, 62 BVMOs were selected for phylogenetic tree construction. As can be seen from Figure 5, the topology of the unrooted phylogenetic tree showed similar clusters with those defined before [117]. The protein MO5660 was assigned to the EthA-like group.

### 3.6. Heterologous Expression and Function Verification of MO5660

**Figure 6:**
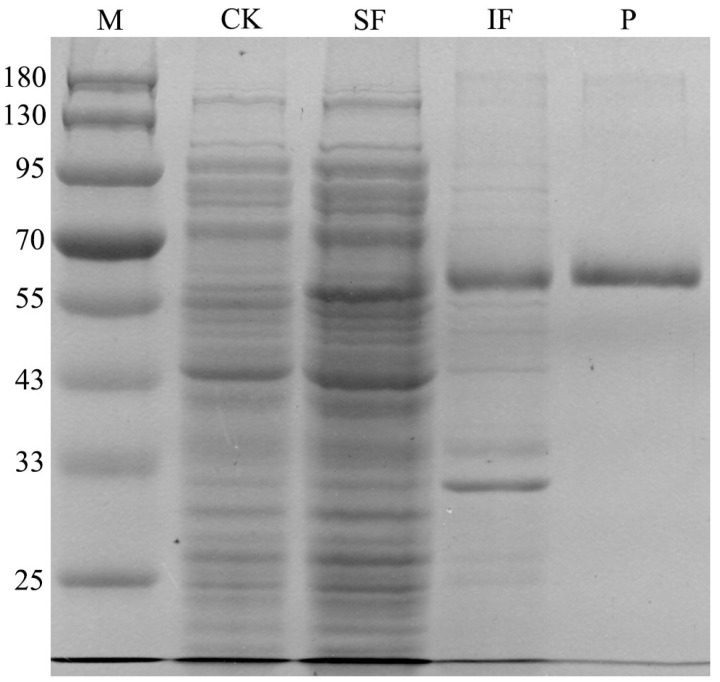
Expression and purification of MO5660. Samples were subjected to 10% SDS-PAGE followed by Coomassie Blue staining. M: Trelief™ Prestained Protein Ladder (Tsingke Biotech Co., Ltd., Beijing, China), CK: Whole protein of pET28a-BL21 (DE3), SF: soluble fractions of the induced culture, IF: insoluble fractions of the induced culture, P: The purified MO5660.

To detect the prometryne-degrading ability of the protein (MO5660) encoded by the *mo5660* gene, this gene was amplified, cloned into *E. coli* BL21(DE3). After induction, the full-length MO5660 protein was produced as a fusion to an N-terminal His-Tag with an expected size of 57.5 kDa (Figure 6). A band of approximated 60 kDa was observed both in the soluble and insoluble fractions of the induced culture. This band was not detected in *E. coli* BL21(DE3)-pET28a cells, which were used as the control (Figure 6, lanes 3 and 4). Subsequently, the His-tagged recombinant protein was purified from the crude extract using Ni-NTA agarose affinity chromatography. After adding 10 μM of FAD to the purified MO5660, HPLC analysis confirmed that the gene engineering strain could degrade prometryne.

### 3.7. Degrading Characteristics of MO5660

**Figure 7:**
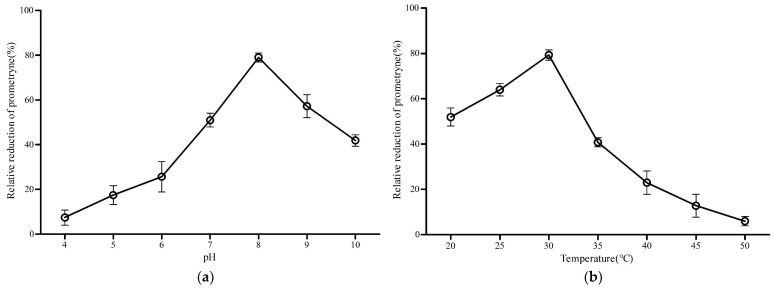
Characterization of the biochemical properties of purified MO5660. (**a**) Effect of pH on MO5660 activity; (**b**) Effect of temperature on MO5660 activity. The error bar indicates one standard deviation.

Figure 7 presents the results obtained from the preliminary analysis of the effect of temperature and pH on the prometryne-degrading activity of MO5660, with prometryne as substrate. As shown in Figure 7a, MO5660 reached its maximum prometryne-degrading activity at pH 8.0 and exhibited more than 50% of its optimal activity at pH 7.0 and 9.0. It can be seen from Figure 7b that the enzyme was active at 20–40 °C with an optimum temperature of 30 °C.

### 3.8. Confirmation of mo5660-Defective Mutation

**Figure 8:**
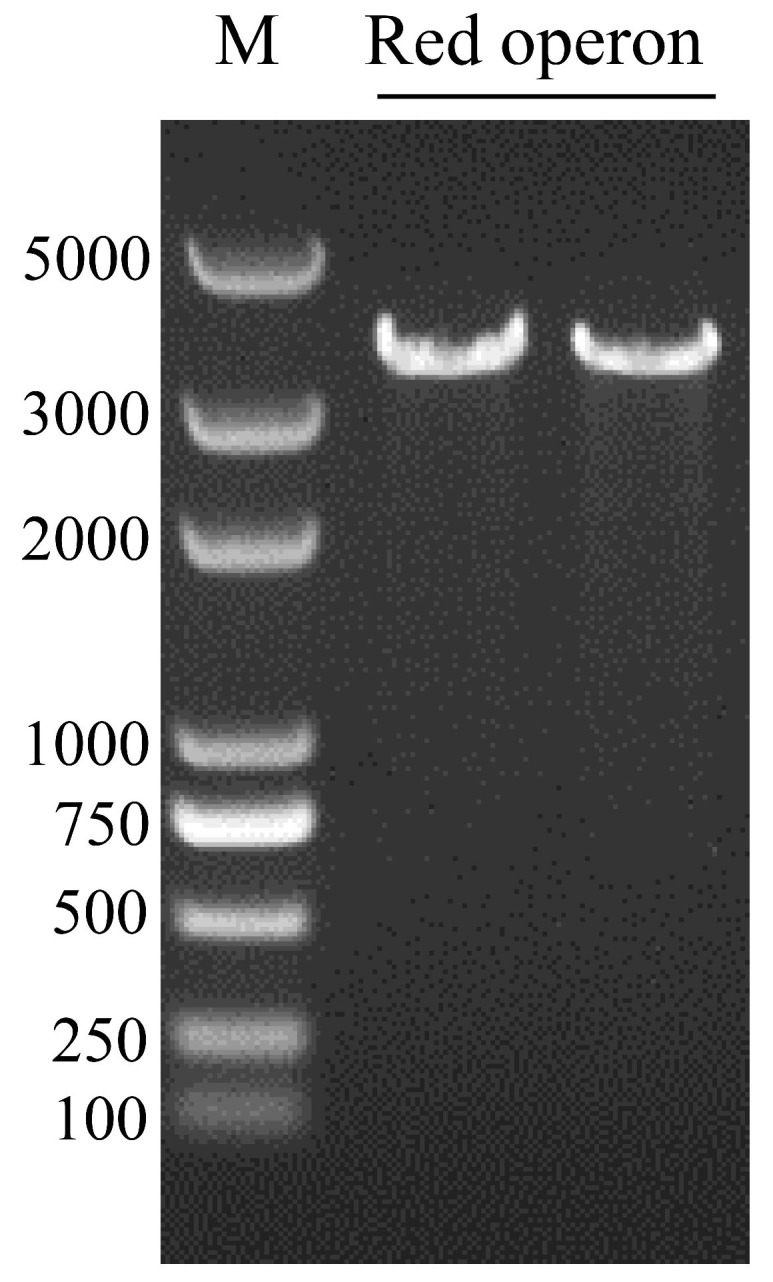
Verification of the Red operon fragment by PCR. M: DL5000 DNA Marker. Red operon indicates the amplified Red operon fragment from pKD46 plasmid with RedF and RedR primers.

To begin this process, a 3393 bp fragment (Figure 8) containing λ-Red and L-arabinose operons was amplified to construct the pUCP18-Red plasmid (Figure S5). The P_BAD_ (*araBAD*) promoter on plasmid pUCP18-Red could be induced by L-arabinose, and then the λ-Red proteins could be expressed efficiently, endowing the DY-1/pUCP18-Red cells with recombination capability.

**Figure 9:**
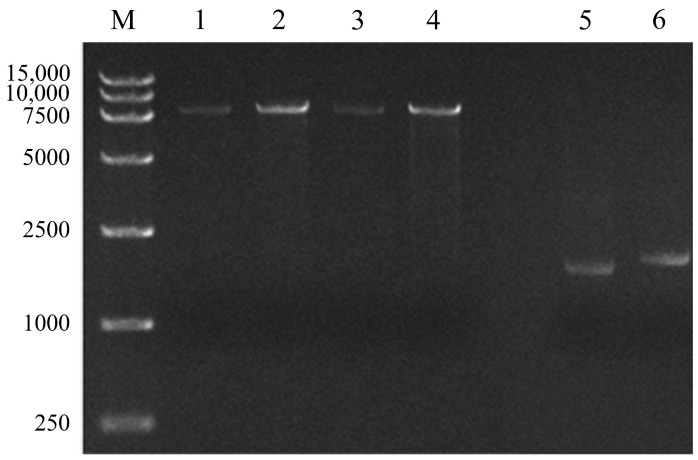
Verification of the recombinant plasmid pUCP18-Red and Δ*mo5660* mutant by enzyme digestion and PCR, respectively. M: DL15,000 DNA Marker. Lanes 1–4: Verification of the Red operon by *Hind* III digestion. Lanes 5 and 6: Verification of gene deletion using two primer pairs outF/kanRin and kanFin/outR.

After electroporating, digestion of the recombinant plasmid extracted from the DY-1/pUCP18-Red colony was carried out with *Hind* III, generated an expected band of around 7.9 kb (Figure 9, Lanes 1–4). After then, the fragment consisted of the upstream region, *kanR*, and downstream region was obtained, purified, and electroporated into DY-1/pUCP18-Red. The *mo5660* gene was then deleted by the integration of *kanR*, which was confirmed with PCR with the non-mutant DY-1/pUCP18-Red (wild type) as the control.

In Figure 9, lanes 5 and 6, PCR products of 1720 and 1749 bp were obtained from Δ*mo5660* genomic DNA with outF/kanRin and kanFin/outR primers, respectively. Six hundred base pairs of upstream and downstream regions of *kanR* were sequenced, results showed that the flanking sequences were identical to the original, and the target gene was defected in the recombinant strain.

**Figure 10:**
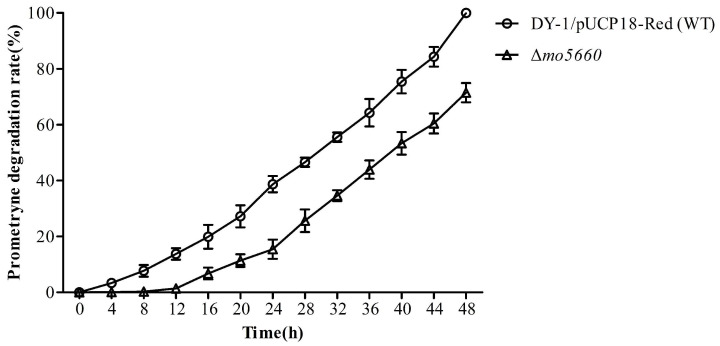
Degradation rates of prometryne by strains WT and Δ*mo5660*. The error bar indicates one standard deviation.

The Δ*mo5660* was then cultured in the prometryne-containing MSM medium to detect the concentration of prometryne after 48 h, with the wild DY-1/pUCP18-Red as a control. As it shows in Figure 10, in the first 12 h, the concentration of reduced prometryne could be hardly detected in the experimental group. However, the mutant performed a degradation rate similar to those by the WT after culturing for 16 h and retained 71.64% of the wild-type activity after 48 h incubation, suggesting that the gene encoding monooxygenase MO5660 was involved in prometryne degradation, but not essential for this process under the laboratory conditions.

## 4. Discussion

The use of herbicides correlates with an improvement in agricultural yield, but the harm to the environment and human health [118]. Prometryne, one of the s-triazine herbicides widely used in China, is a ubiquitous environmental pollutant in water and soil [119]. Microorganisms that have a role in the chloro-s-triazine herbicides metabolic process have been studied extensively [120,121,122], which is in contrast to that for the methylthio-s-triazines. Recently, one of the prometryne-degrading strains, *Leucobacter triazinivorans* JW-1, has got a complete genome which was sequenced and released to the NCBI genome database (NZ_CP035806.1). However, it has been reported that the reaction that happened during the process of degradation of prometryne by JW-1 was deamination [15] rather than oxidation, which occurred during that process of DY-1. Given that, the availability of the complete genome sequence of *Pseudomonas* sp. DY-1 will not only contribute to enriching the genome database but also give us the opportunity to investigate more genes about prometryne degradation and environmental restoration.

In the previous study, DY-1 was identified as a member of the *Pseudomonas* genus based on the alignment of the 16S rRNA gene sequence [20]. However, gene sequences of 16S rRNA hold a low resolution at the intrageneric level [31]. MLSA is a phylogenetic analysis method based on the housekeeping core genes which are highly conservative in phylogeny, and the method has been one of the most acceptable for the phylogenetic assignation of *Pseudomonas* strains [123]. Given that, a phylogenetic tree was constructed based on the results of MLSA, and it was consistent with the published phylogenetic tree [123]. It can be seen from the phylogenetic tree that DY-1 is most closely related to *P. lalkuanensis* PE08, however the similarity not able to reach the cut-off value of the same species, DY-1 can only be determined to belong to *P. resinovorans* phylogenetic group [123].

“Grossly oligotrophic” has been used to describe the soil environment [124]. Therefore, bacteria inhabiting in soil should possess a diverse metabolism to fulfill their nutritional requirements [59]. Rhizosphere microbial communities usually show high species richness. Among these microorganisms, PGPR, which capable of improving plant growth and inhibiting plant pathogens, are beneficial to the sustainable development of agriculture [125]. *Pseudomonas* strains have been widely studied as an effective strategy for biological control [126] they can be used as biological fertilizer, biological control agent, and be used for bioremediation [127]. The results of gene mining show that the genome of DY-1 contains genes involved in signal transduction, carbohydrates metabolism, sulfur metabolism, and adaptation in fluctuating environments, which reveals the potential of DY-1 to promote plant growth and enhance plant resistance. Genome analysis of DY-1 also revealed the presence of genes that might involve in the degradation of various organic pollutants (Tables S11 and S12). While the efficiency is limited when organic pollutants only degraded by bacteria, the efficiency could be higher if bacteria combine with plants, since the rhizosphere secretions of the latter may contribute to the energy metabolism of bacteria [128]. This implicates that DY-1 with the plant-growth-promoting potential may play a greater role in bioremediation. A further study could assess the positive effects of DY-1.

From the genome annotation, we noted that DY-1 did not possess triazine-degrading enzymes coding gene (*atzA* and *trzN*), suggesting that the strain may have another metabolic system. It was previously shown that DY-1 could degrade prometryne and prometryne was first transformed to the corresponding sulfoxide and then to the corresponding sulfone [20]. Since both products had 2-position oxidation suggests that the monooxygenases or/and dioxygenases involved in this process were present in the DY-1 genome. By screening the whole genome, one gene encoding an FMNH_2_-dependent monooxygenase was identified and named *mo5660*. It showed 52.67% identity with EthA, one of the BVMOs, identified in *M. tuberculosis* H37Rv [115]. Through multiple sequence alignment of the protein sequences of MO5660 with other BVMOs, an NADH and a FAD-binding region and two BVMO fingerprint motifs were identified (Figure S4). A phylogenetic tree was constructed, and the results showed that MO5660 shared a high sequence identity with the BVMOs from the EthA-like cluster (Figure 5). These results indicate that MO5660 might have methylthio-oxidation ability. To determine whether *mo5660* was involved in prometryne degradation, heterologous expression was performed. We found that the purified enzyme lost its activity which recovered upon addition of FAD. This phenomenon could be attributed to the loss of the cofactor [129,130,131]. The optimal temperature for MO5660 to degrade prometryne was 30 °C. However, incubation at temperatures above 30 °C resulted in almost 50% loss of its optimal degrading activity, which might be due to its poor stability at high temperatures. A similar characteristic has been obtained from AmBVMO (*Aeromicrobium marinum*) and BoBVMO (*Bradyrhizobium oligotrophicum*), these two BVMOs displayed an instability at temperatures above 30 °C [132]. The optimum pH of MO5660 is slightly alkaline (pH 8.0), which is general among BVMOs [132,133,134]. Using prometryne as substrate, the decrease of its concentration was observed, confirming the degrading role of MO5660. However, gene knockout mediated by λ-Red recombinases system showed that *mo5660* was not essential for prometryne degradation. A possible explanation for this might be that the SSI proteins, for instance, the products of *tauABCD*, *cysPTWA*, and *msuDE*, are involved in the process of prometryne degraded by DY-1. These proteins were not synthesized until cells were grown with alternative sulfur sources such as glutathione or prometryne rather than sulfate or cysteine [135]; thus, the Δ*mo5660* did not show an obvious degradation activity at the first 10 h after the reaction beginning. Given that, the question raised by this study is that there might be another gene, or a gene cluster that exists in DY-1 and play the most important role during degradation. Further research is required to screen more genes responsible for this degradation process so as to understand the complete degrading mechanisms.

## 5. Conclusions

The investigation of the genome of DY-1 confirmed the presence of crucial genes encoding a wide range of mechanisms determining plant growth promotion, rhizosphere colonization, and environmental adaptation, which implied that DY-1 could contribute to promotion of plant growth. Genes involved in PHA synthesis and aromatic compounds degradation are also presented in the DY-1 genome, which revealed the potential of DY-1 for application in the synthesis of environment-friendly compounds and bioremediation of contaminated sites. The second major finding was that a BVMO encoded by *mo5660* in DY-1 did play a role in prometryne degradation with a considerable degradation rate. The result of gene knockout suggested that this enzyme contributes to the improvement of DY-1 degradation efficiency, despite it is not essential for this process. These findings provide new insights into the mechanism of prometryne biodegradation. Several candidate genes (SSI genes) were also identified in the present study, their roles in bioremediation of prometryne-contaminated soil need to be further explored.

## Figures and Tables

**Table 1:** Features of *Pseudomonas* sp. DY-1 genome.

Features	Chromosome	Plasmid
Genome size (bp)	5,886,398	26,350
GC content (mol %)	62.96	56.80
Total genes	5501	42
Total CDS	5410	42
Number of rRNAs (5S, 16S, 23S)	16 (6,5,5)	0
Number of tRNAs	71	0
Number of ncRNAs	4	0

## Supplementary Materials

The following are available online at https://www.mdpi.com/article/10.3390/microorganisms9061261/s1, Table S1: Primers used in this study, Table S2: Selected protein sequences for the analyses of phylogenetic relationships of MO5660, Table S3: Average nucleotide identity based on BLAST (ANIb), Table S4: Genes related to carbohydrate metabolism in DY-1, Table S5: Genes related to nitrogen metabolism in DY-1, Table S6: Genes related to sulfur metabolism in DY-1, Table S7: Genes related to plant grow promoting in DY-1, Table S8: Genes related to environmental stress resistance in DY-1, Table S9: Genes related to heavy metal or metalloid resistance in DY-1, Table S10: Genes related to poly(3-hydroxyalkanoate) biosynthesis in DY-1, Table S11: Genes involved in xenobiotics biodegradation pathways in DY-1, Table S12: Genes related to xenobiotics biodegradation in DY-1, Figure S1: Collinearity analysis of *Pseudomonas* sp. DY-1 genome against *Pseudomonas lalkuanensis* PE08 and *Pseudomonas resinovorans* NBRC 106553 genomes. (a) *Pseudomonas* sp. DY-1 genome and *Pseudomonas lalkuanensis* PE08 genome, (b) *Pseudomonas* sp. DY-1 genome and *Pseudomonas resinovorans* NBRC 106553 genome, Figure S2: Annotation statistics of COG categories of *Pseudomonas* sp. DY-1, Figure S3: Pathway classification of *Pseudomonas* sp. DY-1 genome annotated by KEGG, Figure S4: Protein sequence alignment of MO5600. MO5600: putative FAD-dependent monooxygenase of *Pseudomonas* sp. DY-1 (WP_120651018.1); EthA: Ethionamide monooxygenase from *Mycobacterium tuberculosis* H37Rv (NP_218371.1); BVMO-pp: Baeyer-Villiger monooxygenase from *Pseudomonas putida* KT2440 (WP_176604165.1); AmBVMO: BVMO from *Aeromicrobium marinum* (WP_007076782.1); BoBVMO: BVMO from *Bradyrhizobium oligotrophicum* (WP_015665598.1). The same residues were shown in red, residues with same physico-chemical properties were shown in yellow. The two Rossmann fold motifs (blue), two BVMO fingerprints (orange), and five fingerprints of EthA-like monooxygenase (purple) are marked in different colors, Figure S5: Map of plasmid pUPC18-Red. The backbone was originated from *E. coli*–*P. aeruginosa* shuttle vector pUCP18. Expression of λ-Red genes (*exo*, *bet* and *gam*) driven by P_BAD_ (*araBAD*) promoter are regulated by repressor AraC.
