# Supplementary material for: Complete Genome Sequence and Function Gene Identify of Prometryne-Degrading Strain Pseudomonas sp. DY-1
Source: Microorganisms. 2021 Jun 10;9(6):1261. doi: 10.3390/microorganisms9061261 (PMC8230428; doi:10.3390/microorganisms9061261)

Table S1. Primers used in this study

Name	Sequence (5'→3')
5660F28	CCGAATTCGAGCTCCGTCGACATGACCGCCCAATCGAATC*
5660R28	GTGGTGGTGGTGGTGCTCGAGGGACGCGCCGTCCAGCGC*
RedF	CCC**AAGCTT**ATGACAACTTGACGGCTAC
RedR	CGG**GGTACC**TTCTTCGTCTGTTTCTAC
Fu	CTGGCAATCGCATCGGCGACCACAGGCACCCGCGGGCATG
kanRu	GAAGCAGCTCCAGCCTACACCGAAGCCGAAGGTATAGAGGTCGGAATCCGAGCGGATGCC
kanFd	GGACCATGGCTAATTCCCATGGCCGGGCACGCCCTGGGTGATGTCCATGGATTACTTC
Rd	GCCCTGCGTTTGTCGCCAACGACCTTCGGTGTATTCGATG
kanF	GTGTAGGCTGGAGCTGCTTC
kanR	ATGGGAATTAGCCATGGTCC
kanFin	GGGCACAACAGACAATCGGC
kanRin	GAAGGCGATGCGCTGCGAATCG
outF	AGCCGGTGTGCGGCATGTTCTC
outR	GCTGGGTGCCACCCGTTACC

* Bold nucleotides denote the restriction sites of *Kpn* I and *Hind* III
* Underlined nucleotides are the homology arms to kanamycin resistance gene and pET28a

Table S2. Selected protein sequences for the analyses of phylogenetic relationships of MO5660

Name	Accession number	Strain
EthA	NP_218371.1	*Mycobacterium tuberculosis* H37Rv
BVMO-Pp	AAN68413.1	*Pseudomonas putida* KT2440
AmBVMO	WP_007076782.1	*Aeromicrobium marinum*
BoBVMO	WP_015665598.1	*Bradyrhizobium oligotrophicum*
SMO	BAF48129.1	*Rhodococcus jostii* RHA1
MO1	WP_011598503.1	*Rhodococcus jostii* RHA1
MO2	WP_011596748.1	*Rhodococcus jostii* RHA1
MO3	WP_011595904.1	*Rhodococcus jostii* RHA1
MO4	WP_011595728.1	*Rhodococcus jostii* RHA1
MO5	WP_011594937.1	*Rhodococcus jostii* RHA1
MO6	WP_011594791.1	*Rhodococcus jostii* RHA1
MO7	WP_011598061.1	*Rhodococcus jostii* RHA1
MO9	WP_011599755.1	*Rhodococcus jostii* RHA1
MO10	WP_011599759.1	*Rhodococcus jostii* RHA1
MO11	WP_009479916.1	*Rhodococcus jostii* RHA1
MO12	WP_011598837.1	*Rhodococcus jostii* RHA1
MO14	WP_011596069.1	*Rhodococcus jostii* RHA1
MO15	WP_011595218.1	*Rhodococcus jostii* RHA1
MO17	WP_011597431.1	*Rhodococcus jostii* RHA1
MO19	WP_011597700.1	*Rhodococcus jostii* RHA1
MO20	WP_011599078.1	*Rhodococcus jostii* RHA1
MO21	WP_007295824.1	*Rhodococcus jostii* RHA1
MO24	WP_011597519.1	*Rhodococcus jostii* RHA1
CHMO-Rs	BAH56677.1	*Rhodococcus* sp. HI-31
CPDMO	BAN84077.1	*Pseudomonas* sp. HI-70
PAMO	AAZ55526.1	*Thermobifida fusca* YX
STMO	BAA24454.1	*Rhodococcus rhodochrous* IFO3338
CPMO	CAD10798.1	*Comamonas* sp.NCIMB 9872
CDMO	AAL14233.1	*Rhodococcus ruber* SC1
ACMO	BAF43791.1	*Gordonia* sp. TY-5
HAPMO-Pf	AAK54073.1	*Pseudomonas fluorescens* ACB
HAPMO-Ch	CCF38445.1	*Colletotrichum higginsianum* IMI 349063
OTEMO	AEZ35248.1	*Pseudomonas putida* ATCC 17453
GAMMCHMO	BAA86293.1	*Acinetobacter* sp.NCIMB 9871
PockeMO	AEO56645.1	*Myceliophthora thermophila*
YMOA	CAG79412.1	*Yarrowia lipolytica* CLIB122
CHMO-Bs	AAG01289.1	*Brevibacterium* sp.HCU
CHMO-Phi1	AAN37494.1	*Rhodococcus* sp. Phi1
CHMO-Phi2	AAN37491.1	*Rhodococcus* sp. Phi2

Table S2. Selected protein sequences for the analyses of phylogenetic relationships of MO5660. Continued

Name	Accession number	Strain
CHMO-Pf	AAC36351.2	*Pseudomonas fluorescens* DSM 50106
CHMO-Xf	CAD10801.1	*Xanthobacter flavus*
CHMO-Aa	MBE1536989.1	*Actinomadura algeriensis* DSM 46744
CHMO-Th	RII03651.1	*Thermobifida halotolerans* DSM 44931
CHMO-Nd	GGO75165.1	*Nocardioides deserti* CGMCC4.7183
CHMO-Sc1	AUX34267.1	*Sorangium cellulosum* So ce836
CHMO-Sc2	KYF66511.1	*Sorangium cellulosum* So0003-19-2
CHMO-Sc3	KYG08197.1	*Sorangium cellulosum* So0007-03
CHMO-Mb	ATB32989.1	*Melittangium boletus* DSM 14713
CHMO-Pa	GEL17266.1	*Pseudonocardia asaccharolytica* DSM 44247
BVMOATR8	KFA70082.1	*Stachybotrys chlorohalonata* IBT 40285
BVMO1	CAB55657.1	*Streptomyces coelicolor* A3[2]
BVMO2	CAB59668.1	*Streptomyces coelicolor* A3[2]
BVMO-AFL838	EED54385.1	*Aspergillus flavus* AFL838
BVMO-AFL210	EED54071.1	*Aspergillus flavus* AFL210
BVMO4	AGY78320.1	*Dietzia* sp. D5
MEKMO	ABI15711.1	*Pseudomonas veronii* MEK700
BVMO-Mt	CCP45858.1	*Mycobacterium tuberculosis* H37Rv
2,5-DKCMO	Q6STM1.1	*Pseudomonas putida* ATCC 17453
BVMO-Pa	WP_176604165.1	*Pseudomonas aeruginosa* PAO1
BVMO-Ac	EAW09116.1	*Aspergillus clavatus* NRRL1
BVMO-Pl	ABS63399.1	*Parvibaculum lavamentivorans* DS-1
BVMO-Sa	BAC70705.1	*Streptomyces avermitilis* NBRC 14893
MO5660	WP_120651018.1	*Pseudomonas* sp. DY-1

Figure S1. Collinearity analysis of *Pseudomonas* sp. DY-1 genome against *Pseudomonas lalkuanensis* PE08 and *Pseudomonas resinovorans* NBRC 106553 genomes. **(a)** *Pseudomonas* sp. DY-1 genome and *Pseudomonas lalkuanensis* PE08 genome, **(b)** *Pseudomonas* sp. DY-1 genome and *Pseudomonas resinovorans* NBRC 106553 genome

(a)

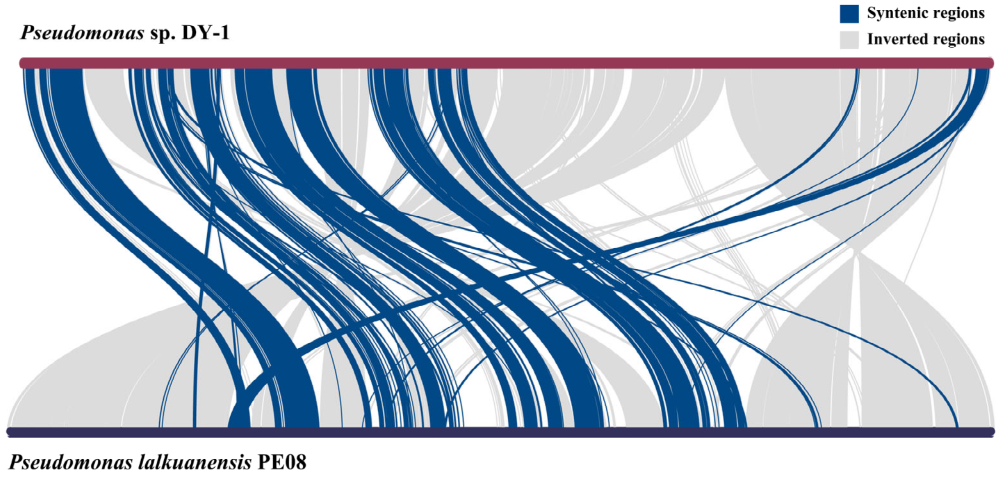

(b)

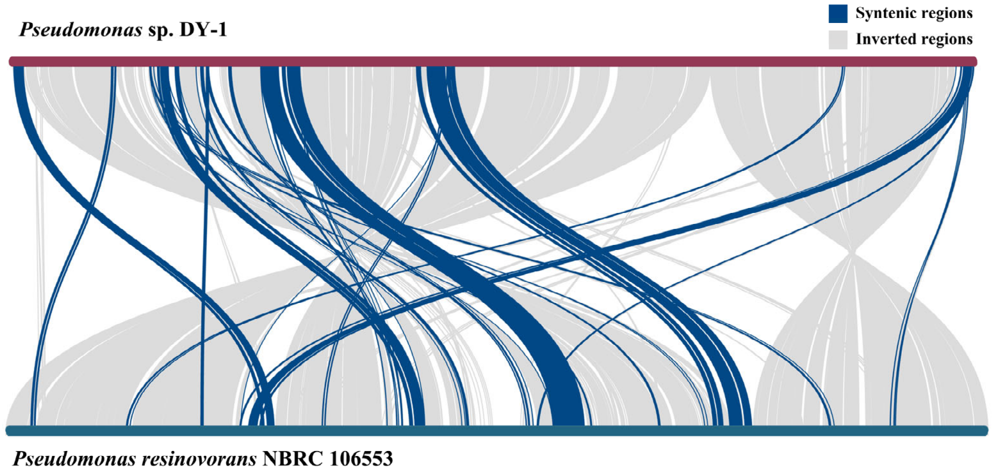

Table S3. Average nucleotide identity based on BLAST (ANIb)

ANIb	DY-1	PE08	NBRC 106553	KF707	DSM 50071	B13	KCTC 32246	KCTC 32247	NCTC 10897
Pseudomonas sp. DY-1	*	87.12	85.83	83.37	76.88	77.11	76.58	76.39	76.38
Pseudomonas lalkuanensis PE08	86.90	*	86.76	84.22	77.71	77.93	77.28	77.03	76.91
Pseudomonas resinovorans NBRC 106553	85.52	86.36	*	84.48	77.97	78.19	77.40	77.30	77.12
Pseudomonas furukawaii KF707	83.28	84.19	84.64	*	77.73	77.53	77.42	77.10	77.16
Pseudomonas aeruginosa DSM 50071	76.91	77.74	78.22	77.71	*	79.59	76.24	77.26	75.84
Pseudomonas knackmussii B13	76.99	78.01	78.38	77.53	79.34	*	76.22	76.96	76.09
Pseudomonas sihuiensis KCTC 32246	76.92	77.75	77.94	77.90	76.54	76.66	*	77.31	87.89
Pseudomonas oryzae KCTC 32247	77.20	78.05	78.56	78.26	78.14	78.10	77.70	*	77.39
Pseudomonas mendocina NCTC 10897	76.68	77.45	77.71	77.59	76.29	76.60	87.87	77.00	*

Figure S2. Annotation statistics of COG categories of *Pseudomonas* sp. DY-1.

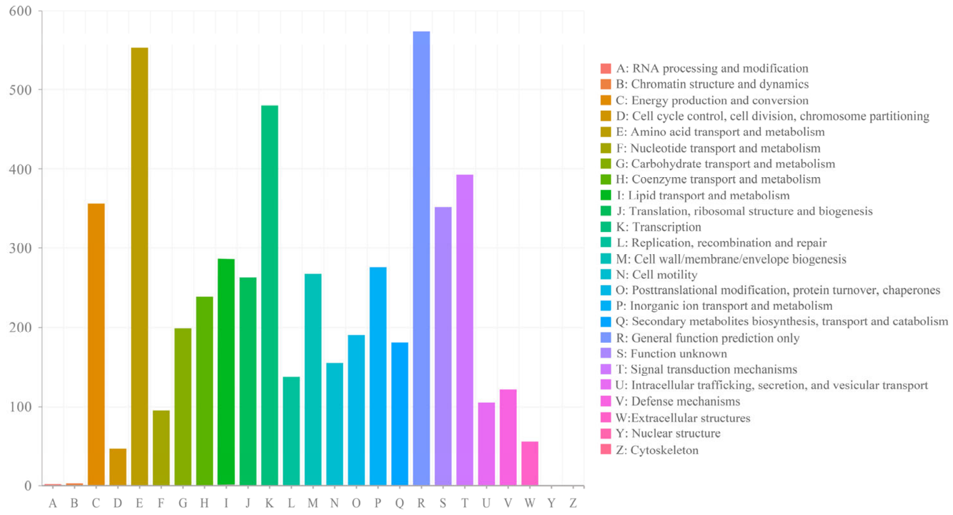

Figure S3. Pathway classification of *Pseudomonas* sp. DY-1 genome annotated by KEGG.

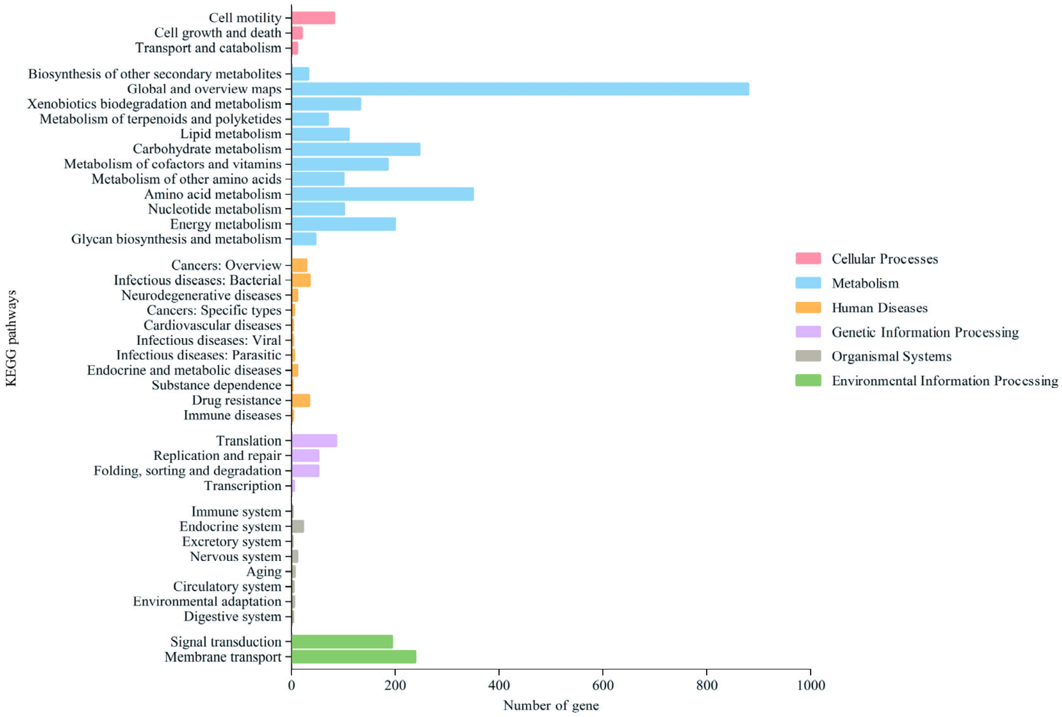

Table S4. Genes related to carbohydrate metabolism in DY-1

Function	Locus tag	Gene	Product	EC code
Pentose phosphate pathway	D6Z43_RS20395,<br>D6Z43_RS27315	*zwf*	glucose-6-phosphate 1-dehydrogenase	1.1.1.49<br>1.1.1.363
	D6Z43_RS03475	*talA*<br>*talB*	transaldolase B	2.2.1.2
	D6Z43_RS04505	*gntK*	gluconokinase	2.7.1.12
	D6Z43_RS09995,<br>D6Z43_RS27325	*eda*	2-dehydro-3-deoxyphosphogluconate aldolase/(4S)-4-hydroxy-2-oxoglutarate aldolase	4.1.2.14<br>4.1.3.42
	D6Z43_RS12220	*phnN*	ribose 1,5-bisphosphokinase	2.7.4.23
	D6Z43_RS17160	*rpe*	ribulose-phosphate 3-epimerase	5.1.3.1
	D6Z43_RS18310	*fbaA*	fructose-bisphosphate aldolase, class II	4.1.2.13
	D6Z43_RS18335	*tktA*<br>*tktB*	transketolase	2.2.1.1
	D6Z43_RS18570	*rpiA*	ribose 5-phosphate isomerase A	5.3.1.6
	D6Z43_RS20750	*algC*	phosphomannomutase/phosphoglucomutase	5.4.2.8<br>5.4.2.2
	D6Z43_RS24115	*pgi*	glucose-6-phosphate isomerase	5.3.1.9
	D6Z43_RS24590	*prs*	ribose-phosphate pyrophosphokinase	2.7.6.1
	D6Z43_RS27260	*edd*	phosphogluconate dehydratase	4.2.1.12
	D6Z43_RS27320	*devB*	6-phosphogluconolactonase	3.1.1.31
	D6Z43_RS27330	*gapN*	glyceraldehyde-3-phosphate dehydrogenase ($NADP^+$)	1.2.1.9
Gluconeogenesis	D6Z43_RS00720,<br>D6Z43_RS09195,<br>D6Z43_RS11100	*gapA*	glyceraldehyde 3-phosphate dehydrogenase	1.2.1.12
	D6Z43_RS07065	*mdh*	malate dehydrogenase	1.1.1.37
	D6Z43_RS22055	*maeB*	malate dehydrogenase (oxaloacetate-decarboxylating) ($NADP^+$)	1.1.1.40
	D6Z43_RS21340	*pckA*	phosphoenolpyruvate carboxykinase (ATP)	4.1.1.49
	D6Z43_RS02700	*pps*	pyruvate, water dikinase	2.7.9.2
	D6Z43_RS26075	*eno*	enolase	4.2.1.11
	D6Z43_RS21670	*gpmI*	2,3-bisphosphoglycerate-independent phosphoglycerate mutase	5.4.2.12
	D6Z43_RS18325	*pgk*	phosphoglycerate kinase	2.7.2.3
	D6Z43_RS21800	*fbp*	fructose-1,6-bisphosphatase I	3.1.3.11
	D6Z43_RS23990	*tpiA*	triosephosphate isomerase (TIM)	5.3.1.1
Entner-Doudoroff pathway	D6Z43_RS10485,<br>D6Z43_RS25445	*pyk*	pyruvate kinase	2.7.1.40
	D6Z43_RS27265	*glk*	glucokinase	2.7.1.2

Table S4. Genes related to carbohydrate metabolism in DY-1. Continued

Function	Locus tag	Gene	Product	EC code
TCA cycle	D6Z43_RS02210	*acnA*	aconitate hydratase	4.2.1.3
	D6Z43_RS02805	*acnB*	aconitate hydratase 2/2-methylisocitrate dehydratase	4.2.1.3<br>4.2.1.99
	D6Z43_RS07175,<br>D6Z43_RS07180	*icd*	isocitrate dehydrogenase	1.1.1.42
	D6Z43_RS08985	*sucD*	succinyl-CoA synthetase α subunit	6.2.1.5
	D6Z43_RS08990	*sucC*	succinyl-CoA synthetase β subunit	6.2.1.5
	D6Z43_RS08995	*pdhD*	dihydrolipoamide dehydrogenase	1.8.1.4
	D6Z43_RS09000	*sucB*	2-oxoglutarate dehydrogenase E2 component	2.3.1.61
	D6Z43_RS09005	*sucA*	2-oxoglutarate dehydrogenase E1 component	1.2.4.2
	D6Z43_RS09010	*sdhB*<br>*frdB*	succinate dehydrogenase/fumarate reductase, iron-sulfur subunit	1.3.5.1<br>1.3.5.4
	D6Z43_RS09015	*sdhA*<br>*frdA*	succinate dehydrogenase/fumarate reductase, flavoprotein subunit	1.3.5.1<br>1.3.5.4
	D6Z43_RS09020	*sdhD*<br>*frdD*	succinate dehydrogenase/fumarate reductase, membrane anchor subunit	-
	D6Z43_RS09025	*sdhC*<br>*frdC*	succinate dehydrogenase/fumarate reductase, cytochrome B subunit	-
	D6Z43_RS09030	*gltA*	citrate synthase	2.3.3.1
Glyoxylate cycle	D6Z43_RS13015	*fumC*	fumarate hydratase, class II	4.2.1.2
	D6Z43_RS25425	*fumA*<br>*fumB*	fumarate hydratase, class I	4.2.1.2
	D6Z43_RS07225	*aceA*	isocitrate lyase	4.1.3.1
Shikimate pathway	D6Z43_RS08175,<br>D6Z43_RS08465,<br>D6Z43_RS16660	*aroF*<br>*aroG*<br>*aroH*	3-deoxy-7-phosphoheptulonate synthase	2.5.1.54
	D6Z43_RS04210,<br>D6Z43_RS06825,<br>D6Z43_RS22950	*aroQ*	3-dehydroquinate dehydratase II	4.2.1.10
	D6Z43_RS06830,<br>D6Z43_RS06840,<br>D6Z43_RS20000,<br>D6Z43_RS27335	*aroE*	shikimate dehydrogenase	1.1.1.25
	D6Z43_RS22090	*aroK*<br>*aroL*	shikimate kinase	2.7.1.71
	D6Z43_RS22095	*aroB*	3-dehydroquinate synthase	4.2.3.4
	D6Z43_RS01835	-	cyclohexadieny/prephenate dehydrogenase/3-phosphoshikimate 1-carboxyvinyltransferase	1.3.1.43<br>1.3.1.12<br>2.5.1.19

Table S4. Genes related to carbohydrate metabolism in DY-1. Continued

Function	Locus tag	Gene	Product	EC code
Shikimate pathway	D6Z43_RS02610	*aroC*	chorismate synthase	4.2.3.5

Table S5. Genes related to nitrogen metabolism in DY-1

Locus tag	Gene	Product	EC code
D6Z43_RS01005	*nirB*	nitrite reductase (NADH) large subunit	1.7.1.4
D6Z43_RS01010	*nirD*	nitrite reductase (NADH) small subunit	1.7.1.4
D6Z43_RS02750	*nasD*	nitrite reductase (NADH)	1.7.1.4
D6Z43_RS02760	*nasA*	MFS transporter, NNP family, nitrate/nitrite transporter	-
D6Z43_RS02795, D6Z43_RS27645, D6Z43_RS27655	*nasF*	nitrate/nitrite transport system substrate-binding protein	-
D6Z43_RS03025	*napA*	nitrate reductase (cytochrome)	1.9.6.1
D6Z43_RS05970	*focA*	formate/nitrite transporter FocA, FNT family	-
D6Z43_RS06950, D6Z43_RS17085	*npd*	nitronate monooxygenase	1.13.12.16
D6Z43_RS09330, D6Z43_RS12780	*glpD*	glycerol-3-phosphate dehydrogenase	1.1.5.3
D6Z43_RS12605	-	ABC-type nitrate/sulfonate/bicarbonate transport system substrate-binding protein	-
D6Z43_RS12610, D6Z43_RS19125, D6Z43_RS19130	-	ABC-type nitrate/sulfonate/bicarbonate transport system permease protein	-
D6Z43_RS12615, D6Z43_RS19120	-	ABC-type nitrate/sulfonate/bicarbonate transport system ATP-binding protein	-
D6Z43_RS12730	*nasC*	assimilatory nitrate reductase catalytic subunit	-
D6Z43_RS14530	*petC*	ubiquinol-cytochrome c reductase cytochrome c1 subunit	-
D6Z43_RS14535	*petB*	ubiquinol-cytochrome c reductase cytochrome b subunit	-
D6Z43_RS14540	*petA*	ubiquinol-cytochrome c reductase iron-sulfur subunit	7.1.1.8
D6Z43_RS18115	*fdsD*	formate dehydrogenase-O, subunit Δ	1.17.1.9
D6Z43_RS18120, D6Z43_RS23135	*fdoG*	formate dehydrogenase-O, subunit α	1.17.1.9
D6Z43_RS18125, D6Z43_RS23140	*fdoH*	formate dehydrogenase-O, iron-sulfur subunit	1.17.1.9
D6Z43_RS18130, D6Z43_RS23145	*fdoI*	formate dehydrogenase-O, subunit γ	
D6Z43_RS23130	*fdnG*	formate dehydrogenase-N	1.17.5.3
D6Z43_RS23150	*fdhE*	FdhE protein	
D6Z43_RS23155	*selA*	L-seryl-tRNA (Ser) seleniumtransferase	2.9.1.1
D6Z43_RS23160	*selB*	selenocysteine-specific elongation factor	
D6Z43_RS27635	*nasD*	nitrate/nitrite transport system ATP-binding protein	7.3.2.4
D6Z43_RS27640	*nasE*	nitrate/nitrite transport system permease protein	-
D6Z43_RS27650	*nasT*	two-component system, response regulator/RNA-binding antiterminator	-

Table S5. Genes related to nitrogen metabolism in DY-1. Continued

Locus tag	Gene	Product	EC code
D6Z43_RS04000	*nac*	LysR family transcriptional regulator, nitrogen assimilation regulatory protein	-
D6Z43_RS04280	*mauD*	methylamine utilization protein	-
D6Z43_RS04285	*mauE*	methylamine utilization protein	-
D6Z43_RS10210	*dmmC*	dimethylamine monooxygenase subunit C	1.14.13.238
D6Z43_RS10215	*dmmB*	dimethylamine monooxygenase subunit B	1.14.13.238
D6Z43_RS10220	*dmmA*	dimethylamine monooxygenase subunit A	1.14.13.238
D6Z43_RS14155	*nifU*	nitrogen fixation protein NifU and related proteins	-
D6Z43_RS19885, D6Z43_RS19890	*can*	carbonic anhydrase	-
D6Z43_RS20900	*glnK*	nitrogen regulatory protein P-II 2	-

Table S6. Genes related to sulfur metabolism in DY-1

Function	Locus tag	Gene	Product	EC code
Organic sulfur	D6Z43_RS01100	*msuD*	$FMNH_2$-dependent methanesulfonate monooxygenase	1.14.14.5
	D6Z43_RS01105	*msuE*	FMN reductase	1.5.1.38
	D6Z43_RS01650, D6Z43_RS19330	*cysP*	sulfate ABC transporter substrate-binding protein	-
	D6Z43_RS08145	*cysB*	LysR family transcriptional regulator, cys regulon transcriptional activator	-
	D6Z43_RS14600, D6Z43_RS26915	*cysD*	sulfate adenylyltransferase subunit 2	2.7.7.4
	D6Z43_RS15530	*astA*	periplasmic sulfonate-binding protein	-
	D6Z43_RS15540	*sftP*	putative TonB-dependent receptor protein	-
	D6Z43_RS15545	*sftR*	LysR family transcriptional regulator	-
	D6Z43_RS19085	*tauA*	taurine ABC transporter substrate binding protein TauA	-
	D6Z43_RS19090	*tauB*	taurine ABC transporter ATP binding protein TauB	-
	D6Z43_RS19095	*tauC*	taurine ABC transporter permease TauC	-
	D6Z43_RS19100	*tauD*	taurine dioxygenase	1.14.11.17
	D6Z43_RS19340	*cysW*	sulfate transporter CysW	-
	D6Z43_RS19335	*cysT*	sulfate transporter CysT	-
	D6Z43_RS19345	*cysA*	sulfate transport system ATP-binding protein	3.6.3.25
	D6Z43_RS19995	*sulP*	sulfate permease	-
	D6Z43_RS24710	*sulP*	sulfate permease	-
	D6Z43_RS24950	*cysZ*	sulfate transporter CysZ	-
Inorganic sulfur	D6Z43_RS22450	*sseA*	thiosulfate/3-mercaptopyruvate sulfurtransferase	2.8.1.1<br>2.8.1.2
	D6Z43_RS03050	*cysI*	sulfite reductase (NADPH) hemoprotein β-component	1.8.1.2
	D6Z43_RS14910	*cysJ*	sulfite reductase (NADPH) flavoprotein α-component	1.8.1.2
	D6Z43_RS02135	*safE*	sulfite exporter SafE	-
	D6Z43_RS24280	*yedY*	sulfite oxidase subunit YedY	-
	D6Z43_RS24285	*yedZ*	sulfite oxidase subunit YedZ	-
	D6Z43_RS02355	*sufS*	cysteine desulfurase/selenocysteine lyase	2.8.1.7<br>4.4.1.16
	D6Z43_RS11805, D6Z43_RS11840, D6Z43_RS19815	*metI*	D-methionine transport system permease protein	-
	D6Z43_RS11810, D6Z43_RS19810	*metN*	D-methionine transport system ATP-binding protein	-
	D6Z43_RS11815, D6Z43_RS11835, D6Z43_RS19820	*metQ*	D-methionine transport system substrate-binding protein	-

Table S7. Genes related to plant grow promoting in DY-1

Function	Locus tag	Gene	Product	EC code
Plant growth promotion	D6Z43_RS02785	-	aromatic-L-amino-acid decarboxylase	4.1.1.28
	D6Z43_RS04565	*amiE*	amidase	3.5.1.4
	D6Z43_RS06715	*aldH*	aldehyde dehydrogenase (NAD^+)	1.2.1.3
	D6Z43_RS09675	-	1-aminocyclopropane-1-carboxylate deaminase	3.5.99.7
	D6Z43_RS13500, D6Z43_RS16555	*ribBA*	3,4-dihydroxy 2-butanone 4-phosphate synthase/GTP cyclohydrolase II	4.1.99.12 3.5.4.25
	D6Z43_RS15240	*ribF*	riboflavin kinase/FMN adenylyltransferase	2.7.1.26 2.7.7.2
	D6Z43_RS16520	*ribA*	GTP cyclohydrolase II	3.5.4.25
	D6Z43_RS16550	*ribH*	6,7-dimethyl-8-ribityllumazine synthase	2.5.1.78
	D6Z43_RS16560	*ribE*	riboflavin synthase	2.5.1.9
	D6Z43_RS16565	*ribD*	diaminohydroxyphosphoribosylaminopyrimidine deaminase/5-amino-6-(5-phosphoribosylamino) uracil reductase	3.5.4.26 1.1.1.193
Volatiles	D6Z43_RS05085, D6Z43_RS18565	*ilvA*	threonine dehydratase	4.3.1.19
	D6Z43_RS05090, D6Z43_RS24255	*ilvB* *ilvG* *ilvI*	acetolactate synthase I/II/III large subunit	2.2.1.6
	D6Z43_RS13510	-	2,3-butanediol dehydrogenase	-
	D6Z43_RS18925	*ilvD*	dihydroxy-acid dehydratase	4.2.1.9
	D6Z43_RS22225	*ilvE*	branched-chain amino acid aminotransferase	2.6.1.42
	D6Z43_RS24260	*ilvH* *ilvN*	acetolactate synthase I/III small subunit	2.2.1.6
	D6Z43_RS24265	*ilvY*	LysR family transcriptional regulator, positive regulator for *ilvC*	-
	D6Z43_RS24270	*ilvC*	ketol-acid reductoisomerase	1.1.1.86
Phosphate solubilization	D6Z43_RS01795, D6Z43_RS02275, D6Z43_RS17155, D6Z43_RS19920	*gph*	phosphoglycolate phosphatase	3.1.3.18
	D6Z43_RS03855	*pqqF*	pyrroloquinoline quinone biosynthesis protein F	-
	D6Z43_RS03860	*pqqE*	PqqA peptide cyclase	1.21.98.4
	D6Z43_RS03865	*pqqD*	pyrroloquinoline quinone biosynthesis protein D	-
	D6Z43_RS03870	*pqqC*	pyrroloquinoline-quinone synthase	1.3.3.11
	D6Z43_RS03875	*pqqB*	pyrroloquinoline quinone biosynthesis protein B	-
	D6Z43_RS03880	*pqqA*	pyrroloquinoline quinone biosynthesis protein A	-
	D6Z43_RS07195	*mupP*	N-acetyl-D-muramate 6-phosphate phosphatase	3.1.3.105
	D6Z43_RS07200	*ubiG*	2-polyprenyl-6-hydroxyphenyl methylase/3-demethylubiquinone-9 3-methyltransferase	2.1.1.222/2.1.1.64
	D6Z43_RS16535	*pgpA*	phosphatidylglycerophosphatase A	3.1.3.27

Table S7. Genes related to plant grow promoting in DY-1. Continued

Function	Locus tag	Gene	Product	EC code
	D6Z43_RS20995	*gmhB*	D-glycero-D-manno-heptose 1,7-bisphosphate phosphatase	3.1.3.82<br>3.1.3.83
	D6Z43_RS22210	*pdhC*	pyruvate dehydrogenase E2 component (dihydrolipoamide acetyltransferase)	2.3.1.12
	D6Z43_RS23470	*phnA*	phosphonoacetate hydrolase	3.11.1.2
	D6Z43_RS01795, D6Z43_RS02275, D6Z43_RS17155, D6Z43_RS19920	*gph*	phosphoglycolate phosphatase	3.1.3.18
	D6Z43_RS03855	*pqqF*	pyrroloquinoline quinone biosynthesis protein F	-
	D6Z43_RS03860	*pqqE*	PqqA peptide cyclase	1.21.98.4
Phosphate solubilization	D6Z43_RS03865	*pqqD*	pyrroloquinoline quinone biosynthesis protein D	-
	D6Z43_RS03870	*pqqC*	pyrroloquinoline-quinone synthase	1.3.3.11
	D6Z43_RS03875	*pqqB*	pyrroloquinoline quinone biosynthesis protein B	-
	D6Z43_RS03880	*pqqA*	pyrroloquinoline quinone biosynthesis protein A	-
	D6Z43_RS07195	*mupP*	N-acetyl-D-muramate 6-phosphate phosphatase	3.1.3.105
	D6Z43_RS07200	*ubiG*	2-polyprenyl-6-hydroxyphenyl methylase/3-demethylubiquinone-9 3-methyltransferase	2.1.1.22<br>2.1.1.64
	D6Z43_RS16535	*pgpA*	phosphatidylglycerophosphatase A	3.1.3.27
	D6Z43_RS20995	*gmhB*	D-glycero-D-manno-heptose 1,7-bisphosphate phosphatase	3.1.3.82<br>3.1.3.83
	D6Z43_RS22210	*pdhC*	pyruvate dehydrogenase E2 component (dihydrolipoamide acetyltransferase)	2.3.1.12
	D6Z43_RS23470	*phnA*	phosphonoacetate hydrolase	3.11.1.2
	D6Z43_RS07750, D6Z43_RS08320, D6Z43_RS14620, D6Z43_RS15540	-	TonB-dependent siderophore receptor	-
Pathogen inhibition (Iron acquisition)	D6Z43_RS12170, D6Z43_RS21195	*fbpA*	iron (III) transport system substrate-binding protein	-
	D6Z43_RS12175, D6Z43_RS21200	*fbpB*	iron (III) transport system permease protein	-
	D6Z43_RS12180	*fbpC*	iron (III) transport system ATP-binding protein	3.6.3.30
	D6Z43_RS21060	*efeU*	high-affinity Fe^{2+}/Pb^{2+} permease	-
	D6Z43_RS23910	*fur*	Fur family transcriptional regulator, ferric uptake regulator	-

Table S8. Genes related to environmental stress resistance in DY-1

Function	Locus tag	Gene	Product	EC code
Tolerance of ROS	D6Z43_RS01170, D6Z43_RS06475, D6Z43_RS04690, D6Z43_RS06540	*osmC*	lipoyl-dependent peroxiredoxin	1.11.1.28
	D6Z43_RS03355	*norR*	anaerobic nitric oxide reductase transcription regulator	-
	D6Z43_RS03360	*fhp*	nitric oxide dioxygenase (EC:1.14.12.17)	-
	D6Z43_RS03370	-	uncharacterized protein involved in response to NO	-
	D6Z43_RS06465	*katG*	catalase-peroxidase	1.11.1.21
	D6Z43_RS06470	-	formamides	3.5.1.49
	D6Z43_RS07405	*katE*	catalase	1.11.1.6
	D6Z43_RS08375	*gst*	glutathione S-transferase	2.5.1.18
	D6Z43_RS08380	*msrC*	L-methionine (R)-S-oxide reductase	1.8.4.14
	D6Z43_RS08390	*sagS*	two-component system, sensor histidine kinase SagS	2.7.13.3
	D6Z43_RS08395, D6Z43_RS08505, D6Z43_RS27065, D6Z43_RS27255, D6Z43_RS27340	*ohrR*	MarR family transcriptional regulator, organic hydroperoxide resistance regulator	-
	D6Z43_RS13265	*ahpF*	alkyl hydroperoxide reductase	-
	D6Z43_RS08710, D6Z43_RS13270, D6Z43_RS26845	*ahpC*	lipoyl-dependent peroxiredoxin subunit C	1.11.1.28
	D6Z43_RS00460, D6Z43_RS26710	*ahpD*	alkylhydroperoxidase AhpD	-
	D6Z43_RS08400	*gpx*	glutathione peroxidase	1.11.1.9
	D6Z43_RS08405	*msrB*	peptide-methionine (R)-S-oxide reductase	1.8.4.12
	D6Z43_RS08410	*alaA*	alanine-synthesizing transaminase	2.6.1.66 2.6.1.2
	D6Z43_RS26225	*gst*	glutathione S-transferase	2.5.1.18
	D6Z43_RS26245	*cybB*	superoxide oxidase	1.10.3.17
	D6Z43_RS27095	*kefB*	glutathione-regulated potassium-efflux system protein KefB	-

Table S8. Genes related to environmental stress resistance in DY-1. Continued

Function	Locus tag	Gene	Product	EC code
Alginate biosynthesis （Exopolysaccharides）	D6Z43_RS05855, D6Z43_RS13940	*algA*	mannose-1-phosphate guanylyltransferase/mannose-6-phosphate isomerase	2.7.7.13 5.3.1.8
	D6Z43_RS05860, D6Z43_RS13935	*algF*	alginate O-acetyltransferase complex protein AlgF	-
	D6Z43_RS05865	*algJ*	alginate O-acetyltransferase complex protein AlgJ	-
	D6Z43_RS05870, D6Z43_RS13925	*algI*	alginate O-acetyltransferase complex protein AlgI	-
	D6Z43_RS05875, D6Z43_RS13920	*algL*	poly(β-D-mannuronate) lyase	4.2.2.3
	D6Z43_RS05880, D6Z43_RS13915	*algX*	alginate biosynthesis protein AlgX	-
	D6Z43_RS05885, D6Z43_RS13910	*algG*	mannuronan 5-epimerase	5.1.3.37
	D6Z43_RS05890, D6Z43_RS13905	*algE*	alginate production protein	-
	D6Z43_RS05895, D6Z43_RS13900	*algK*	alginate biosynthesis protein AlgK	-
	D6Z43_RS05900, D6Z43_RS13895	*alg44*	mannuronan synthase	2.4.1.33
	D6Z43_RS05905, D6Z43_RS13890	*alg8*	mannuronan synthase	2.4.1.33
	D6Z43_RS05910, D6Z43_RS13885	*algD*	GDP-mannose 6-dehydrogenase	1.1.1.132
	D6Z43_RS12435	*algU*	RNA polymerase sigma factor AlgU	-
	D6Z43_RS13930	*algV*	alginate biosynthesis protein AlgV	-
	D6Z43_RS14615	*algW*	AlgW protein	-
	D6Z43_RS20750	*algC*	phosphomannomutase	-
	D6Z43_RS20990	*algZ*	two-component system, LytTR family, sensor histidine kinase AlgZ	2.7.13.3
	D6Z43_RS20995	*algR*	two-component system, LytTR family, response regulator AlgR	-
	D6Z43_RS21030	*algQ*	anti-RNA polymerase sigma 70 factor	-
	D6Z43_RS21035	*algP*	alginate regulatory protein	-

Table S8. Genes related to environmental stress resistance in DY-1. Continued

Function	Locus tag	Gene	Product	EC code
Cold shock response	D6Z43_RS00985, D6Z43_RS07170, D6Z43_RS08555, D6Z43_RS11310, D6Z43_RS14220, D6Z43_RS25900, D6Z43_RS26460	*cspA*	cold shock protein	-
Universal stress response	D6Z43_RS02815, D6Z43_RS06220, D6Z43_RS11285	*uspE*	universal stress protein E	-
	D6Z43_RS09285	*uspA*	universal stress protein A	-
	D6Z43_RS13000	*bolA*	BolA family transcriptional regulator, general stress-responsive regulator	-
pH adaptation and osmoregulation	D6Z43_RS00680	*phaA* *phaB*	pH adaptation potassium efflux system protein A/B	-
	D6Z43_RS00685	*phaC*	pH adaptation potassium efflux system protein C	-
	D6Z43_RS00690	*phaD*	pH adaptation potassium efflux system protein D	-
	D6Z43_RS00695	*mrpE*	multiple resistance and pH homeostasis protein E	-
	D6Z43_RS00700	*phaF*	pH adaptation potassium efflux system protein F	-
	D6Z43_RS00705	*phaG*	pH adaptation potassium efflux system protein G	-
	D6Z43_RS00810, D6Z43_RS17990, D6Z43_RS18175	*gbdR*	AraC family transcriptional regulator, glycine betaine-responsive activator	-
	D6Z43_RS02025	*aauR*	two-component system, response regulator AauR	-
	D6Z43_RS02030	*aauS*	two-component system, sensor histidine kinase AauS	2.7.13.3
	D6Z43_RS02970	*nhaB*	sodium/proton antiporter	-
	D6Z43_RS03800, D6Z43_RS04945	*bcct*	choline/carnitine/betaine transport protein	-
	D6Z43_SR03810	*dctA3*	Na^+/H^+-dicarboxylate symporter, C4-dicarboxylate transport protein 3	-
	D6Z43_RS06370	*nhaA*	Na^+/H^+ antiporter NhaA	-
	D6Z43_RS08430	*DOPA*	4,5-DOPA dioxygenase extradiol	1.13.11.-
	D6Z43_RS10625	*opuE*	solute:Na^+ symporter, SSS family	-
	D6Z43_RS10995	*dctA2*	Na^+/H^+-dicarboxylate symporter	-
	D6Z43_RS11590, D6Z43_RS15650, D6Z43_RS18225, D6Z43_RS26795	*betB*	betaine-aldehyde dehydrogenase	1.2.1.8
	D6Z43_RS14060	*mscM*	miniconductance mechanosensitive channel	-

Table S8. Genes related to environmental stress resistance in DY-1. Continued

Function	Locus tag	Gene	Product	EC code
pH adaptation and osmoregulation	D6Z43_RS14355	*mscS*	small-conductance mechanosensitive channel	-
	D6Z43_RS15410	*mscL*	large-conductance mechanosensitive channel	-
	D6Z43_RS18035	*gbcB*	glycine betaine catabolism B	-
	D6Z43_RS18045	*gbcA*	glycine betaine catabolism A	-
	D6Z43_RS18095	*cdhA*	carnitine 3-dehydrogenase	1.1.1.108
	D6Z43_RS18105, D6Z43_RS18185, D6Z43_RS19460, D6Z43_RS19990, D6Z43_RS21835, D6Z43_RS21850	*proX*	glycine betaine/proline transport system substrate-binding protein	-
	D6Z43_RS18110	*cdhR*	AraC family transcriptional regulator, carnitine catabolism transcriptional activator	-
	D6Z43_SR18190, D6Z43_RS21860	*proV*	glycine betaine/proline transport system ATP-binding protein	7.6.2.9
	D6Z43_RS18195, D6Z43_RS19455, D6Z43_RS21855	*proW*	glycine betaine/proline transport system permease protein	-
	D6Z43_RS18210, D6Z43_RS18220	*betI*	TetR/AcrR family transcriptional regulator, transcriptional repressor of bet genes	-
	D6Z43_RS18215, D6Z43_RS20885	*betS*	choline/glycine/proline betaine transport protein	-
	D6Z43_RS18230, D6Z43_RS19800	*betA*	choline dehydrogenase	1.1.99.1
	D6Z43_RS19985	*betC*	choline-sulfatase	3.1.6.6
	D6Z43_RS20045	*trkA*	Trk system potassium uptake protein	-
	D6Z43_RS20280, D6Z43_RS21460	*dctD*	two-component system, NtrC family, C4-dicarboxylate transport response regulator	-
	D6Z43_RS20285, D6Z43_RS21465	*dctB*	two-component system, NtrC family, C4-dicarboxylate transport sensor histidine kinase	2.7.13.3
	D6Z43_RS22175	*mscK*	potassium-dependent mechanosensitive channel	-
	D6Z43_RS22180	*nhaP2*	potassium/proton antiporter NhaP2	-
	D6Z43_RS22635	*mscS*	small-conductance mechanosensitive channel	-
	D6Z43_RS23500	*opuE*	solute:Na^+ symporter, SSS family	-
	D6Z43_RS25555	*nhaP*	NhaP-type Na^+/H^+ or K^+/H^+ antiporter	-
	D6Z43_RS27095	*kefB*	glutathione-regulated potassium-efflux system protein KefB	-

Table S8. Genes related to environmental stress resistance in DY-1. Continued

Function	Locus tag	Gene	Product	EC code
Cyanide metabolism	D6Z43_RS07665, D6Z43_RS13110, D6Z43_RS02000	-	CP family, cyanate transporter	-
	D6Z43_RS15455	*cydB*	cytochrome bd ubiquinol oxidase subunit II	7.1.1.7
	D6Z43_RS15460	*cydA*	cytochrome bd ubiquinol oxidase subunit I	7.1.1.7
	D6Z43_RS07590	*mqoA*	malate:quinone oxidoreductase	1.1.5.4
	D6Z43_RS24725	*mqoB*	malate:quinone oxidoreductase	1.1.5.4
	D6Z43_RS20725	*crc*	catabolite repression control protein	-

Table S9. Genes related to heavy metal or metalloid resistance in DY-1

Function	Locus tag	Gene	Product	EC code
Zinc, cadmium, and lead	D6Z43_RS02510	*zupT*	zinc transporter, ZIP family	-
	D6Z43_RS02720	*zntB*	zinc transporter	-
	D6Z43_RS19780	*znuA*	zinc transport system substrate-binding protein	-
	D6Z43_RS19785	*zur*	Fur family transcriptional regulator, zinc uptake regulator	-
	D6Z43_RS19790	*znuC*	zinc transporter ZnuC	-
	D6Z43_RS19795	*znuB*	zinc ABC transporter permease	-
	D6Z43_RS25780	*zntA*	Cd^{2+}/Zn^{2+}-exporting ATPase	3.6.3.3<br>3.6.3.5
	D6Z43_RS25785	*merR*	Cd (II)/Pb (II)-responsive transcriptional regulator	-
Copper and silver	D6Z43_RS02145, D6Z43_RS23350	*copA*	P-type Cu^{+} transporter	7.2.2.8
	D6Z43_RS03425	*copS*	sensor protein CopS	2.7.13.3
	D6Z43_RS03430	*copR*	transcriptional activator protein CopR	-
	D6Z43_RS03540, D6Z43_RS03585	*copB*	copper resistance protein B	-
	D6Z43_RS03550	*cotA*	copper resistance protein A	-
	D6Z43_RS03575, D6Z43_RS23360	*copZ*	copper chaperone CopZ	-
	D6Z43_RS05500, D6Z43_RS06195, D6Z43_RS07050, D6Z43_RS08075, D6Z43_RS11130	-	bilirubin oxidase	1.3.3.5
	D6Z43_RS05795	*cusA*<br>*silA*	copper/silver efflux system protein	-
	D6Z43_RS05800	*cusB*<br>*silB*	membrane fusion protein, copper/silver efflux system	-
	D6Z43_RS05805	*czcC*	outer membrane protein, heavy metal efflux system	-
	D6Z43_RS14025	*mmcO*	multicopper oxidase	1.16.3.1
	D6Z43_RS22660	*azu*	azurin	-
	D6Z43_RS23345	*cueR*	MerR family copper efflux transcriptional regulator	-
Others	D6Z43_RS00930	*tehA*	tellurite resistance protein TehA	-
	D6Z43_RS04955, D6Z43_RS19610	*tehC*	predicted tellurium resistance membrane protein	-
	D6Z43_RS04110	*chrA*	chromate transporter	-
	D6Z43_RS04115	*chrB*	chromate resistance exported protein	-
	D6Z43_RS05770, D6Z43_RS26395	*arsC*	arsenate reductase (thioredoxin)	1.20.4.4
	D6Z43_RS05780	*arsB*	arsenical pump membrane protein	-

Table S9. Genes related to heavy metal or metalloid resistance in DY-1. Continued

Function	Locus tag	Gene	Product	EC code
	D6Z43_RS11105	*arsH*	arsenical resistance protein ArsH	-
Others	D6Z43_RS09475	*amtB*	ammonium transporter, Amt family	-
	D6Z43_RS14625	*cbiK*	nickel transport protein	-

Table S10. Genes related to poly(3-hydroxyalkanoate) biosynthesis in DY-1

Locus tag	Gene	Product	EC code
D6Z43_RS09265	*fadA*	3-ketoacyl-CoA thiolase	2.3.1.16
D6Z43_RS09270	*fadB*	fatty acid oxidation complex subunit α	-
D6Z43_RS12125	*phaJ1*	(R)-specific enoyl-CoA hydratase	-
D6Z43_RS21975	-	putative polyhydroxyalkanoic acid system protein	-
D6Z43_RS21980	*phaI*	polyhydroxyalkanoate synthesis regulator phasin	-
D6Z43_RS21985	*phaF*	polyhydroxyalkanoate synthesis regulator phasin	-
D6Z43_RS21990	-	transcriptional regulator, TetR family	-
D6Z43_RS21995	*phaC2*	poly(3-hydroxyalkanoate) polymerase 2	2.3.1.-
D6Z43_RS22000	*phaZ*	poly(3-hydroxyalkanoate) depolymerase	3.1.1.-
D6Z43_RS22005	*phaC1*	poly(3-hydroxyalkanoate) polymerase 1	2.3.1.-
D6Z43_RS22185	*phaD*	short-chain acyl-CoA dehydrogenase	1.3.8.1

Table S11. Genes involved in xenobiotics biodegradation pathways in DY-1.

Function	Locus tag	Gene	Product	EC code
Homogentisate and aromatic amino acids	D6Z43_RS00885, D6Z43_RS12970	*mai*	maleylacetoacetate isomerase	5.2.1.2
	D6Z43_RS10070	*tyrB*	aromatic-amino-acid transaminase	2.6.1.57
	D6Z43_RS11755	*phhR*	transcriptional regulator PhhR	-
	D6Z43_RS11760	*phhA*	phenylalanine 4-monooxygenase	1.14.16.1
	D6Z43_RS11765	*phhB*	pterin-4-alpha-carbinolamine dehydratase	4.2.1.96
	D6Z43_RS11770	*phhC*	aromatic-amino-acid transaminase	2.6.1.57
	D6Z43_RS11780	*hmgA*	homogentisate 1,2-dioxygenase	1.13.11.5
	D6Z43_RS11785	*fahA*	fumarylacetoacetase	3.7.1.2
β-ketoadipate pathway − the protocatechuate branch	D6Z43_RS05550	*pcaQ*	*pca* operon transcription factor PcaQ	-
	D6Z43_RS05555	*pcaH*	protocatechuate 3,4-dioxygenase subunit β	1.13.11.3
	D6Z43_RS05560	*pcaG*	protocatechuate 3,4-dioxygenase subunit α	-
	D6Z43_RS05580, D6Z43_RS06835	*pcaR*	transcriptional regulator PcaR	-
	D6Z43_RS05585	*pcaK*	4-hydroxybenzoate transporter PcaK	-
	D6Z43_RS06835	*pcaR*	IclR family transcriptional regulator, *pca* regulon regulatory protein	-
	D6Z43_RS06915, D6Z43_RS14815	*pcaC*	4-carboxymuconolactone decarboxylase	4.1.1.44
	D6Z43_RS06920, D6Z43_RS11425	*pcaD*	β-ketoadipate enol-lactone hydrolase II	3.1.1.24
	D6Z43_RS06925	*pcaB*	3-carboxy-cis,cis-muconate cycloisomerase	5.5.1.2
	D6Z43_RS06930	*pcaF*	3-oxoadipyl-CoA thiolase	2.3.1.174
	D6Z43_RS06935	*pcaJ*	β-ketoadipate:succinyl-CoA transferase subunit B	2.3.8.6
	D6Z43_RS06940	*pcaI*	β-ketoadipate:succinyl-CoA transferase subunit A	-
	D6Z43_RS05465	*pobA*	p-hydroxybenzoate 3-monooxygenase	1.14.13.2
	D6Z43_RS05470	*pobR*	AraC family transcriptional regulator, transcriptional activator of *pobA*	-
β-ketoadipate pathway− the catechol branch	D6Z43_RS27420	*catR*	transcriptional regulator	-
	D6Z43_RS27425, D6Z43_RS11150, D6Z43_RS16465	*catA*	catechol 1,2-dioxygenase	1.13.11.1
	D6Z43_RS27430	*catB*	muconate cycloisomerase I	5.5.1.1
	D6Z43_RS27435	*catC*	muconolactone Δ-isomerase	5.3.3.4
Benzoate pathway	D6Z43_RS12840	*xylK*	4-hydroxy-2-oxovalerate/4-hydroxy-2-oxohexanoate aldolase	4.1.3.39<br>4.1.3.43

Table S11. Genes involved in xenobiotics biodegradation pathways in DY-1. Continued

Pathway	Locus tag	Gene	Product	EC code
	D6Z43_RS12845	*xylQ*	acetaldehyde/propanal dehydrogenase	1.2.1.10<br>1.2.1.87
	D6Z43_RS12850	*xylJ*	2-oxopent-4-enoate/cis-2-oxohex-4-enoate hydratase	4.2.1.80<br>4.2.1.132
	D6Z43_RS05590	*xylS3*	*xylDLEGF* operon transcriptional activator 3	-
	D6Z43_RS05595	*xylX*	benzoate/toluate 1,2-dioxygenase α subunit	1.14.12.10<br>1.14.12.-
Benzoate pathway	D6Z43_RS05600	*xylY*	benzoate/toluate 1,2-dioxygenase β subunit	1.14.12.10<br>1.14.12.-
	D6Z43_RS05605	*xylZ*	benzoate/toluate 1,2-dioxygenase reductase subunit	1.18.1.-
	D6Z43_RS05610	*xylL*	1,6-dihydroxycyclohexa-2,4-diene-1-carboxylate dehydrogenase	1.3.1.25<br>1.3.1.-
	D6Z43_RS05615	*benK*	MFS transporter, AAHS family, benzoate transport protein	-
	D6Z43_RS05620	*benE*	benzoate membrane transport protein	-
	D6Z43_RS06485	*xylH*	4-oxalocrotonate tautomerize	5.3.2.6
	D6Z43_RS03905,<br>D6Z43_RS07830,<br>D6Z43_RS07850,<br>D6Z43_RS11945,<br>D6Z43_RS27040	*feaB*	phenylacetaldehyde dehydrogenase	1.2.1.39
	D6Z43_RS07865	*paaX*	phenylacetic acid degradation operon negative regulatory protein	-
	D6Z43_RS07870	*paaY*	phenylacetic acid degradation protein	-
	D6Z43_RS07875	*paaN*	phenylacetic acid degradation protein	-
	D6Z43_RS07880	*paaF*	enoyl-CoA hydratase	4.2.1.17
Phenylacetate pathway	D6Z43_RS07885	*paaG*	2-(1,2-epoxy-1,2-dihydrophenyl) acetyl-CoA isomerase	5.3.3.18
	D6Z43_RS07890	*paaH*	3-hydroxybutyryl-CoA dehydrogenase	1.1.1.157
	D6Z43_RS07895	*paaI*	acyl-CoA thioesterase	3.1.2.-
	D6Z43_RS07900	*paaJ*	β-ketoadipyl-CoA thiolase	2.3.1.174
	D6Z43_RS07905	*paaK*	phenylacetate-CoA ligase	6.2.1.30
	D6Z43_RS08005	*paaA*	ring-1,2-phenylacetyl-CoA epoxidase subunit PaaA	1.14.13.149
	D6Z43_RS08010	*paaB*	ring-1,2-phenylacetyl-CoA epoxidase subunit PaaB	-

Table S11. Genes involved in xenobiotics biodegradation pathways in DY-1. Continued

Pathway	Locus tag	Gene	Product	EC code
Phenylacetate pathway	D6Z43_RS08015	*paaC*	ring-1,2-phenylacetyl-CoA epoxidase subunit PaaC	1.14.13.149
	D6Z43_RS08020	*paaD*	ring-1,2-phenylacetyl-CoA epoxidase subunit PaaD	-
	D6Z43_RS08025	*paaE*	ring-1,2-phenylacetyl-CoA epoxidase subunit PaaE	-
	D6Z43_RS08045	*paaZ*	oxepin-CoA hydrolase / 3-oxo-5,6-dehydrosuberyl-CoA semialdehyde dehydrogenase	3.3.2.12<br>1.2.1.91
4-hydroxyphenylacetate	D6Z43_RS11975	*hpaA*	AraC family transcriptional regulator, 4-hydroxyphenylacetate 3-monooxygenase operon regulatory protein	-
	D6Z43_RS11980,<br>D6Z43_RS11985	*hpaG*	5-carboxymethyl-2-oxo-hex-3-ene-1,7-dioate decarboxylase	4.1.1.68<br>5.3.3.-
	D6Z43_RS11990	*hpaE*	5-carboxymethyl-2-hydroxymuconic-semialdehyde dehydrogenase	1.2.1.60
	D6Z43_RS11995	*hpaD*	3,4-dihydroxyphenylacetate 2,3-dioxygenase	1.13.11.15
	D6Z43_RS12000	*hpaF*	5-carboxymethyl-2-hydroxymuconate isomerase	5.3.3.10
	D6Z43_RS12005	*hpaX*	4-hydroxyphenylacetate symporter, major facilitator superfamily (MFS)	-
	D6Z43_RS12010	*hpaH*	2-oxo-hept-3-ene-1,7-dioate hydratase	4.2.1.-
	D6Z43_RS12015	*hpaI*	4-hydroxy-2-oxoheptanedioate aldolase	4.1.2.52
	D6Z43_RS12020	*hpaC*	4-hydroxyphenylacetate 3-hydroxylase, reductase component	1.5.1.36
	D6Z43_RS12025	*hpaB*	4-hydroxyphenylacetate 3-monooxygenase	1.14.14.9
	D6Z43_RS12030	*hpaR*	homoprotocatechuate degradation operon regulator HpaR	-

Table S12. Genes related to xenobiotics biodegradation in DY-1.

Compound	Locus tag	Gene	Product	EC code
Nitriles	D6Z43_RS17410	*oxdA*	aldoxime dehydratase	4.99.1.5
	D6Z43_RS17415	*nhaA*	nitrile hydratase subunit α	4.2.1.84
	D6Z43_RS17420	*nhaB*	nitrile hydratase subunit β	4.2.1.84
	D6Z43_RS17425	*amiE*	amidase	3.5.1.4
Azo	D6Z43_RS03685, D6Z43_RS11410, D6Z43_RS11735, D6Z43_RS22990	*azoR*	FMN-dependent NADH-azoreductase	1.7.-. -
	D6Z43_RS07325	*azr*	azobenzene reductase	1.7.1.6
	D6Z43_RS21760	*pnbA*	para-nitrobenzyl esterase/Carboxylesterase type B	-
Sodium lauryl sulphate (SDS)	D6Z43_RS12590	*sdsA*	alkyl sulfatase	-
	D6Z43_RS12595	*sdsB*	SDS degradation transcriptional activation protein	-
Straight-chain alkane compounds	D6Z43_RS05170, D6Z43_RS22675	*ALDH*	aldehyde dehydrogenase (NAD^+)	1.2.1.3
	D6Z43_RS06975, D6Z43_RS21085	*dlh*	dienelactone hydrolase	-
	D6Z43_RS11455	*alkB*	alpha-ketoglutarate-dependent dioxygenase AlkB	-
	D6Z43_RS11820, D6Z43_RS11845	*ladA*	long-chain alkane monooxygenase	1.14.14.28
	D6Z43_RS26095	*adhC*	S-(hydroxymethyl)glutathione dehydrogenase / alcohol dehydrogenase	1.1.1.284 1.1.1.1
	D6Z43_RS21090	-	alkylglycerol monooxygenase	1.14.16.5
	D6Z43_RS25290	*oleD*	2-alkyl-3-oxoalkanoate reductase	1.1.1.412
Others	D6Z43_RS09265	*fadA*	3-ketoacyl-CoA thiolase	2.3.1.16
	D6Z43_RS09270	*fadB*	fatty acid oxidation complex subunit alpha	-
	D6Z43_RS12840	*tesG*	4-hydroxy-2-oxovalerate/4-hydroxy-2-oxohexanoate aldolase	4.1.3.39 4.1.3.43
	D6Z43_RS12845	*tesF*	acetaldehyde/propanal dehydrogenase	1.2.1.10 1.2.1.87
	D6Z43_RS12850	*tesE*	2-oxopent-4-enoate/cis-2-oxohex-4-enoate hydratase	4.2.1.80 4.2.1.132
	D6Z43_RS12855	*hsaD*	4,5:9,10-diseco-3-hydroxy-5,9,17-trioxoandrosta-1(10),2-diene-4-oate hydrolase	3.7.1.17
	D6Z43_RS12865	-	bile acid 7-alpha dehydratase	-
	D6Z43_RS12870	*ksi*	steroid delta-isomerase	5.3.3.1
	D6Z43_RS12875	*atoB*	acetyl-CoA acetyltransferase	2.3.1.9

Table S12. Genes related to xenobiotics biodegradation in DY-1. Continued

Compound	Locus tag	Gene	Product	EC code
	D6Z43_RS12890	*fadE30*	acyl-CoA dehydrogenase FadE30	-
	D6Z43_RS12945	*hsaC*	3,4-dihydroxy-9,10-secoandrosta-1,3,5(10)-triene-9,17-dione 4,5-dioxygenase	1.13.11.25
	D6Z43_RS13465	*kstD*	3-oxosteroid 1-dehydrogenase	1.3.99.4
	D6Z43_RS13530	*kshA*	3-ketosteroid 9alpha-monooxygenase subunit A	1.14.15.30
	D6Z43_RS13670	*mpc*	catechol 2, 3-dioxygenase	
	D6Z43_RS05170, D6Z43_RS22675	*aldH*	aldehyde dehydrogenase (NAD^+)	1.2.1.3
	D6Z43_RS13680	*mhpA*	3-(3-hydroxy-phenyl) propionate hydroxylase	1.14.13.127
	D6Z43_RS13830	-	carboxylesterase 2	-
	D6Z43_RS16430	*dmpP*	phenol/toluene 2-monooxygenase (NADH) P5/A5	1.14.13.244<br>1.14.13.243
	D6Z43_RS16435	*dmpO*	phenol/toluene 2-monooxygenase (NADH) P4/A4	1.14.13.244<br>1.14.13.243
	D6Z43_RS16440	*dmpN*	phenol/toluene 2-monooxygenase (NADH) P3/A3	1.14.13.244<br>1.14.13.243
Others	D6Z43_RS16445	*dmpM*	phenol/toluene 2-monooxygenase (NADH) P2/A2	1.14.13.244<br>1.14.13.243
	D6Z43_RS16450	*dmpL*	phenol/toluene 2-monooxygenase (NADH) P1/A1	1.14.13.244<br>1.14.13.243
	D6Z43_RS16455	*dmpK*	phenol/toluene 2-monooxygenase (NADH) P0/A0	-
	D6Z43_RS21515	*mhpE*	4-hydroxy 2-oxovalerate aldolase	4.1.3.39
	D6Z43_RS26870	*hsaA*	3-hydroxy-9,10-secoandrosta-1,3,5(10)-triene-9,17-dione monooxygenase	1.14.14.12
	D6Z43_RS26880	*tesI*	3-oxo-5alpha-steroid 4-dehydrogenase	1.3.99.5
	D6Z43_RS26885	*hsd*	3(or 17) beta-hydroxysteroid dehydrogenase	1.1.1.51
	D6Z43_RS26890	*kshB*	3-ketosteroid 9alpha-monooxygenase subunit B	1.14.15.30
	D6Z43_RS26900	*fadD3*	HIP-CoA ligase	6.2.1.41
	D6Z43_RS27480	*antC*	anthranilate 1,2-dioxygenase reductase subunit	1.18.1.-
	D6Z43_RS27485	*antB*	anthranilate 1,2-dioxygenase (deaminating, decarboxylating) small subunit	1.14.12.1
	D6Z43_RS27490	*antA*	anthranilate 1,2-dioxygenase (deaminating, decarboxylating) large subunit	1.14.12.1

Figure S4. Protein sequence alignment of MO5600. MO5600: putative FAD-dependent monooxygenase of Pseudomonas sp. DY-1 (WP_120651018.1); EthA: Ethionamide monooxygenase from *Mycobacterium tuberculosis* H37Rv (NP_218371.1); BVMO-pp: Baeyer-Villiger monooxygenase from *Pseudomonas putida* KT2440 (WP_176604165.1); AmBVMO: BVMO from *Aeromicrobium marinum* (WP_007076782.1); BoBVMO: BVMO from *Bradyrhizobium oligotrophicum* (WP_015665598.1). The same residues were shown in red, residues with same physico-chemical properties were shown in yellow. The two Rossmann fold motifs (blue), two BVMO fingerprints (orange), and five fingerprints of EthA-like monooxygenase (purple) are marked in different colors.

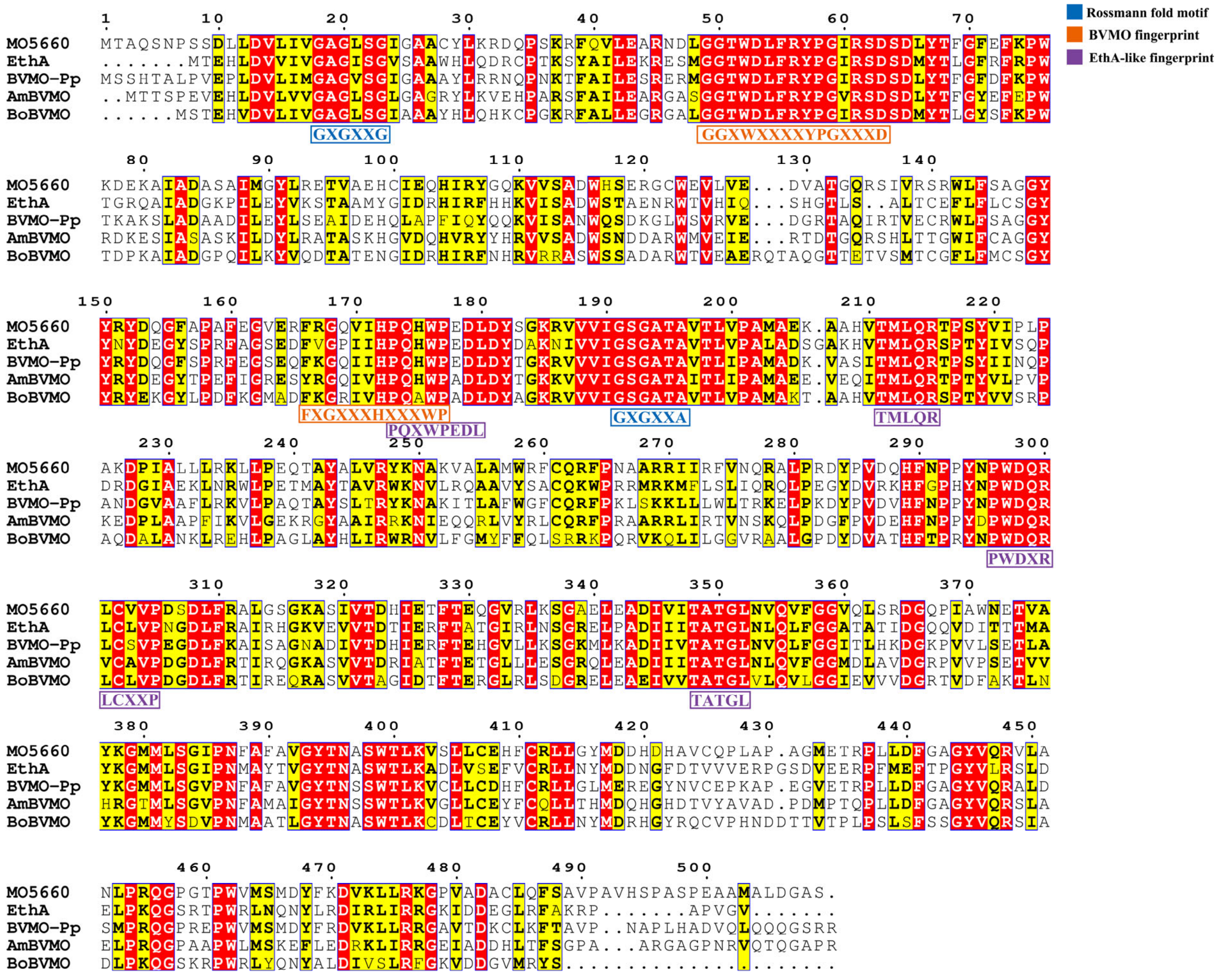

Figure S5. Map of plasmid pUPC18-Red. The backbone was originated from *E. coli*–*P. aeruginosa* shuttle vector pUCP18. Expression of λ-Red genes (*exo*, *bet* and *gam*) driven by PBAD (*araBAD*) promoter are regulated by repressor AraC.

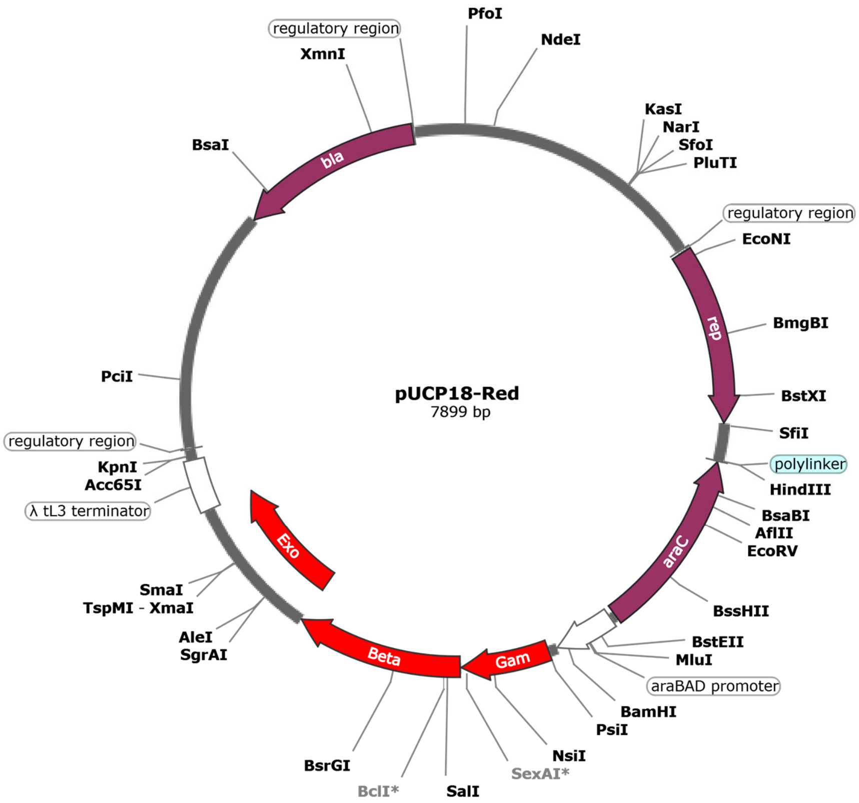

Supplement: Supplementary file 1 [file microorganisms-09-01261-s001.zip › Supplementary_files_revised.pdf]
