# Supplementary figures and images for: Complete Genome Sequence and Function Gene Identify of Prometryne-Degrading Strain Pseudomonas sp. DY-1
Source: Microorganisms. 2021 Jun 10;9(6):1261. doi: 10.3390/microorganisms9061261 (PMC8230428; doi:10.3390/microorganisms9061261)

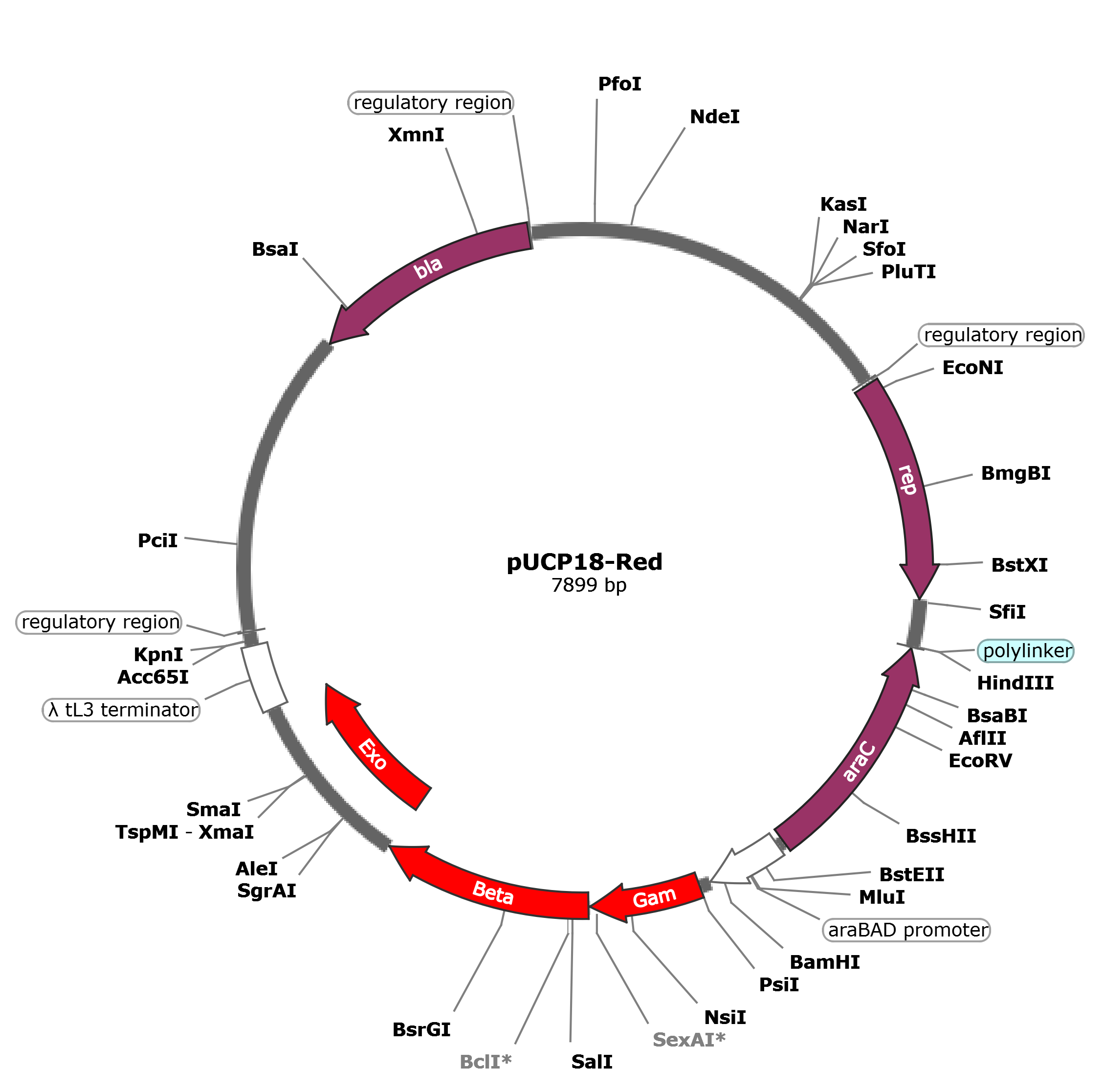

Supplement: Supplementary file 1 [file microorganisms-09-01261-s001.zip › pUCp18-Red_map.png]
